# A Flexible and Accurate Genotype Imputation Method for the Next Generation of Genome-Wide Association Studies

**DOI:** 10.1371/journal.pgen.1000529

**Published:** 2009-06-19

**Authors:** Bryan N. Howie, Peter Donnelly, Jonathan Marchini

**Affiliations:** 1 Department of Statistics, University of Oxford, Oxford, United Kingdom; 2 Wellcome Trust Centre for Human Genetics, Roosevelt Drive, Oxford, United Kingdom; University of California San Diego and The Scripps Research Institute, United States of America

## Abstract

Genotype imputation methods are now being widely used in the analysis of genome-wide association studies. Most imputation analyses to date have used the HapMap as a reference dataset, but new reference panels (such as controls genotyped on multiple SNP chips and densely typed samples from the 1,000 Genomes Project) will soon allow a broader range of SNPs to be imputed with higher accuracy, thereby increasing power. We describe a genotype imputation method (IMPUTE version 2) that is designed to address the challenges presented by these new datasets. The main innovation of our approach is a flexible modelling framework that increases accuracy and combines information across multiple reference panels while remaining computationally feasible. We find that IMPUTE v2 attains higher accuracy than other methods when the HapMap provides the sole reference panel, but that the size of the panel constrains the improvements that can be made. We also find that imputation accuracy can be greatly enhanced by expanding the reference panel to contain thousands of chromosomes and that IMPUTE v2 outperforms other methods in this setting at both rare and common SNPs, with overall error rates that are 15%–20% lower than those of the closest competing method. One particularly challenging aspect of next-generation association studies is to integrate information across multiple reference panels genotyped on different sets of SNPs; we show that our approach to this problem has practical advantages over other suggested solutions.

## Author Summary

Large association studies have proven to be effective tools for identifying parts of the genome that influence disease risk and other heritable traits. So-called “genotype imputation” methods form a cornerstone of modern association studies: by extrapolating genetic correlations from a densely characterized reference panel to a sparsely typed study sample, such methods can estimate unobserved genotypes with high accuracy, thereby increasing the chances of finding true associations. To date, most genome-wide imputation analyses have used reference data from the International HapMap Project. While this strategy has been successful, association studies in the near future will also have access to additional reference information, such as control sets genotyped on multiple SNP chips and dense genome-wide haplotypes from the 1,000 Genomes Project. These new reference panels should improve the quality and scope of imputation, but they also present new methodological challenges. We describe a genotype imputation method, IMPUTE version 2, that is designed to address these challenges in next-generation association studies. We show that our method can use a reference panel containing thousands of chromosomes to attain higher accuracy than is possible with the HapMap alone, and that our approach is more accurate than competing methods on both current and next-generation datasets. We also highlight the modeling issues that arise in imputation datasets.

## Introduction

Genome-wide association studies have identified many putative disease susceptibility loci in recent years [1]–[3]. This approach to studying disease has succeeded largely because of improved catalogues of human genetic variation [4] and advances in genotyping technology, but it has also been bolstered by the rise of genotype imputation methods [5]–[8], which have allowed researchers to tease increasingly subtle signals out of large and complex genetic datasets [9],[10].

**Figure 1:**
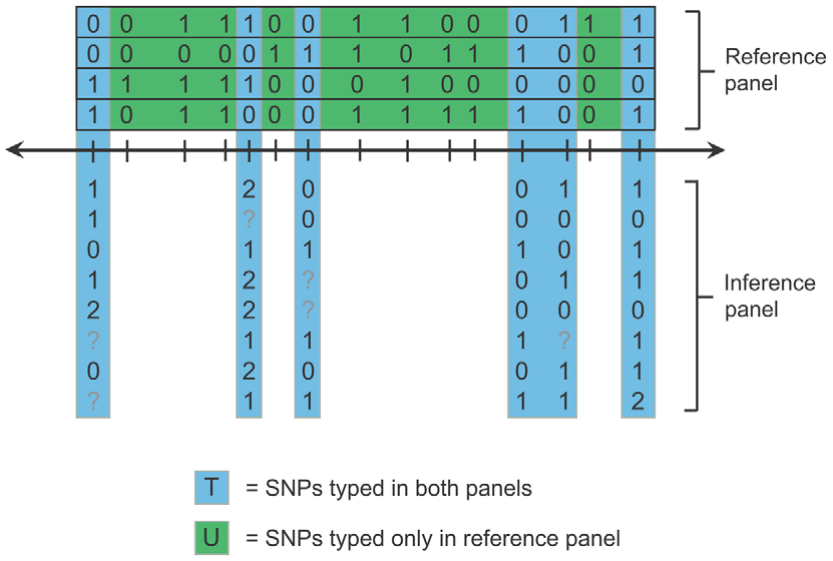
Schematic drawing of imputation Scenario A. In this drawing, haplotypes are represented as horizontal boxes containing 0's and 1's (for alternate SNP alleles), and unphased genotypes are represented as rows of 0's, 1's, 2's, and ?'s (where ‘1’ is the heterozygous state and ‘?’ denotes a missing genotype). The SNPs (columns) in the dataset can be partitioned into two disjoint sets: a set *T* (blue) that is genotyped in all individuals and a set *U* (green) that is genotyped only in the haploid reference panel. The goal of imputation in this scenario is to estimate the genotypes of SNPs in set *U* in the study sample.

Imputation methods work by combining a *reference panel* of individuals genotyped at a dense set of polymorphic sites (usually single-nucleotide polymorphisms, or “SNPs”) with a *study sample* collected from a genetically similar population and genotyped at a subset of these sites. Figure 1 shows a schematic example of such a dataset. Imputation methods predict unobserved genotypes in the study sample by using a population genetic model to extrapolate allelic correlations measured in the reference panel. The imputed genotypes expand the set of SNPs that can be tested for association, and this more comprehensive view of the genetic variation in a study can enhance true association signals and facilitate meta-analysis [9],[10].

To date, most imputation analyses have used reference panels composed of haplotypes from Phase II of the International HapMap Project, together with study samples genotyped on commercial genome-wide SNP arrays. Figure 1 depicts this arrangement, which we call *Scenario A*. To understand how imputation methods work in this setting, it helps to observe that the SNPs exist in a natural hierarchy, such that they can be partitioned into two disjoint sets: a set *T* that is *typed* in both the study sample and the reference panel, and a set *U* that is *untyped* in the study sample but typed in the reference panel. Informally, most imputation methods phase the study genotypes at SNPs in *T* and look for perfect or near matches between the resulting haplotypes and the corresponding partial haplotypes in the reference panel—haplotypes that match at SNPs in *T* are assumed to also match at SNPs in *U*. This is the fundamental basis of genotype imputation.

Several important points emerge from this description. First, the accuracy with which the study haplotypes are phased at SNPs in *T* should determine how well they can be matched to haplotypes in the reference panel, which should in turn influence the accuracy of imputation at SNPs in *U*. Second, accounting for the unknown phase of the SNPs in *T* can be computationally expensive; if the haplotypes at these SNPs were known, most methods would be able to impute genotypes at SNPs in *U* more quickly. Third, many existing methods do not use all of the available information to phase the study genotypes at SNPs in *T*. In principle, a phasing algorithm should be able to “learn” about desirable phasing configurations for a given study individual by pooling information across the reference panel and all other individuals in the study, and the phasing accuracy should increase with the sample size; in standard practice, most imputation methods gain phasing information about each study individual only from the reference panel, and phasing accuracy does not depend on the size of the study sample. (This description applies to imputation methods based on hidden Markov models, or “HMMs” [6],[11]; non-HMM methods often discard other kinds of information.) The BEAGLE imputation model [12],[13] is one notable exception to this point, and we discuss its alternative modeling strategy in detail in this work.

We have developed a new algorithm that seeks to improve imputation accuracy at untyped SNPs by improving phasing accuracy at typed SNPs, building on the points raised above. Most HMM-based imputation methods simultaneously estimate missing genotypes and analytically integrate over the unknown phase of SNPs in *T*. By contrast, we propose to alternately estimate haplotypes at SNPs in *T* and impute alleles at SNPs in *U*, assuming the haplotype guesses are correct. We account for the phasing uncertainty in the data by iterating these steps in a Markov chain Monte Carlo (MCMC) framework. Separating the phasing and imputation steps allows us to focus more computational effort on phasing and use more of the available information; the extra computation used in this step is largely balanced by the quick haploid imputation in the step that follows.

This approach can improve imputation accuracy in Scenario A, as we show in the Results section, but another major motivation of this work is to extend IMPUTE [6] to handle “next-generation” association datasets. By this, we refer to studies in the near future that will have access to additional reference data that could inform imputation. Next-generation reference panels will present new challenges for imputation, including larger sample sizes; unphased and incomplete genotypes; and multiple reference panels containing different SNP sets. Our method aims to use the principles outlined above to address these challenges and improve imputation accuracy in next-generation studies.

**Figure 2:**
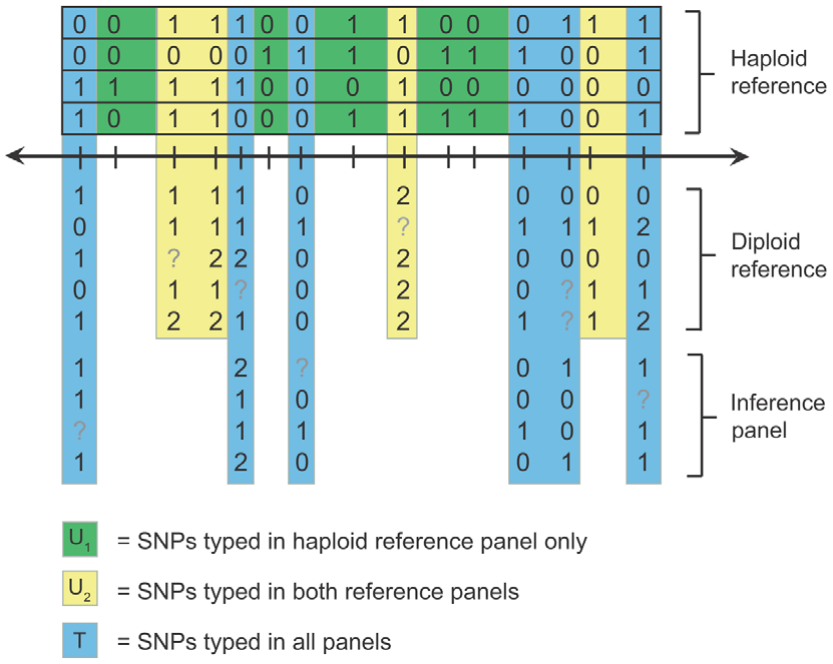
Schematic drawing of imputation Scenario B. In this drawing, haplotypes are represented as horizontal boxes containing 0's and 1's (for alternate SNP alleles), and unphased genotypes are represented as rows of 0's, 1's, 2's, and ?'s (where ‘1’ is the heterozygous state and ‘?’ denotes a missing genotype). The SNPs (columns) in the dataset can be partitioned into three disjoint sets: a set *T* (blue) that is genotyped in all individuals, a set *U_2_* (yellow) that is genotyped in both the haploid and diploid reference panels but not the study sample, and a set *U_1_* (green) that is genotyped only in the haploid reference panel. The goal of imputation in this scenario is to estimate the genotypes of SNPs in set *U_2_* in the study sample and SNPs in the set *U_1_* in both the study sample and, if desired, the diploid reference panel.

One new data configuration, which we call *Scenario B* and explore in detail in the current study, is presented in Figure 2; we will address other next-generation reference panels in the Discussion. In Scenario B, there are different amounts of genotype data in different cohorts of a study. For example, the Wellcome Trust Case Control Consortium (WTCCC) is currently performing an association study in which 6,000 controls will be genotyped on both the Affymetrix 6.0 and Illumina 1 M SNP chips, whereas disease cohorts will be genotyped only on either the Affymetrix 6.0 chip or the Illumina 670 k chip. In other words, a large set of controls will be genotyped at a subset of HapMap SNPs, and each case cohort will be genotyped at a subset of the SNPs typed in the controls. Published studies have already employed this design [14], and it may become more prevalent in the future as common sets of population controls become more widely available.

In Scenario B, the study individuals genotyped on a larger number of SNPs can be used as an unphased, or “diploid”, reference panel for imputation in the remaining samples (which do not necessarily have to be cases). As before, we approach such a dataset by partitioning the SNPs into disjoint sets, named with reference to the study sample: a set *U_1_* that is *untyped* in the study sample and typed only in the haploid reference panel, a set *U_2_* that is *untyped* in the study sample and typed in both the haploid and diploid reference panels, and a set *T* that is *typed* in all samples.

We apply the same inference principles to Scenario B as to Scenario A: at each MCMC iteration we phase all of the observed data, pooling information across samples typed on common sets of SNPs to estimate each haplotype pair, then perform haploid imputation assuming that all of the haplotype guesses are correct. One novelty of this scenario is that, at SNPs in *U_2_*, the reference panel may contain thousands of chromosomes, in contrast to HapMap Phase II panels that contain only 120–180 chromosomes each. In principle, this added depth should improve imputation accuracy at SNPs in *U_2_*, with notable gains at rare SNPs. The latter point is especially relevant because rare SNPs are an important source of power in imputation analyses [5],[6]. Scenario B also introduces the problem of multiple reference panels genotyped on different, hierarchical sets of SNPs. Many next-generation imputation datasets will follow this paradigm, which presents modeling challenges that remain largely unexplored.

In the sections that follow, we describe the details of our new method as applied to the scenarios in Figure 1 and Figure 2. We then compare the method with other imputation approaches on real datasets from the United Kingdom that emulate Scenarios A and B. We show that our method can attain higher accuracy than existing methods in Scenario A, but that the absolute gains are small, which we attribute to the inherent limitations of a small set of reference haplotypes. In an example of Scenario B, we demonstrate that our method can use a large unphased reference panel to achieve higher accuracy than imputation based on the HapMap alone. We also show that our method can impute genotypes more accurately than other sophisticated [11],[13] and simpler [15] methods applied to the same dataset, and that our approach has higher sensitivity and specificity to detect copies of the minor allele at rare SNPs. In addition, we present results that highlight important practical advantages of our imputation modeling strategy over the one used by BEAGLE.

We have implemented our new imputation method as an update to our existing software package IMPUTE; the new program is called “IMPUTE version 2” (IMPUTE v2). We refer to our previously published method [6] as “IMPUTE version 1” (IMPUTE v1).

## Materials and Methods

### Software

IMPUTE v1 and IMPUTE v2 are freely available for academic use from the website http://www.stats.ox.ac.uk/~marchini/software/gwas/gwas.html

### Scenario A

In Scenario A, IMPUTE v2 estimates marginal posterior probabilities of missing genotypes by alternately phasing all of the SNPs in *T* in the study sample (simultaneously imputing any sporadically missing genotypes) and then imputing study genotypes at the SNPs in *U*, conditional on the haplotype guesses from the first step. To explain this process in more detail, we begin by defining 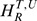, the set of known reference haplotypes at SNPs in *T* and *U* (i.e., the entire reference panel); 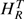, the set of known reference haplotypes at SNPs in *T*; and 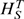, the set of unobserved study haplotypes at SNPs in *T*. If there are *N_S_* individuals in the study sample, their haplotypes at SNPs in *T* can be represented as 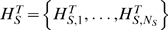, where 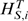 is the haplotype pair for study individual *i*.

The method begins by choosing initial guesses for the haplotypes in 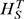 – by default, we choose haplotypes that are consistent with the observed genotype data but phased at random. We then perform a number of MCMC iterations. Each iteration updates every study individual *i* (in some arbitrary order) in two steps:

1. Sample a new haplotype pair 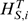 for individual *i* at SNPs in *T*. This is accomplished by sampling from the conditional distribution 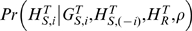, where 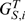 is individual *i*'s multilocus genotype at SNPs in *T*, 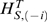 contains current-guess haplotypes at SNPs in *T* for all study individuals except *i*, 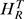 contains the reference panel haplotypes at SNPs in *T*, and *ρ* is the fine-scale, population-scaled recombination map for the region of interest. We describe this distribution further below.
2. Impute new alleles (in two independent haploid steps) for SNPs in *U*, conditional on 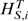, 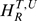, and *ρ*.

We typically run the method for a relatively small number of burn-in iterations that invoke only the phasing step, followed by a larger number of main iterations that include both steps and contribute to the final imputation probabilities. We investigate the convergence properties of the method in Text S1, Figure S1, and Table S1.

In Step 1, the algorithm phases individual *i*'s observed genotype 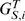 by sampling from 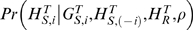. The model we use to specify this conditional distribution is essentially the same one used by IMPUTE v1 [6] – i.e., we use a hidden Markov model that is based on an approximation to the coalescent-with-recombination process [16]. This model views newly sampled haplotypes as “imperfect mosaics” of haplotypes that have already been observed. As with IMPUTE v1, we use an estimated fine-scale recombination map [17] for SNP-to-SNP transition probabilities and a result from population genetics theory [6] for emission probabilities, which model historical mutation.

One difference between versions is that IMPUTE v1 analytically integrates over the unknown phase of the genotypes in the study sample, whereas IMPUTE v2 uses Step 1 to integrate over the space of phase reconstructions via Monte Carlo. This step is accomplished for each individual by sampling a pair of paths through the hidden states (haplotypes) of the model, then probabilistically sampling a pair of haplotypes that is consistent with the observed multilocus genotype. Path sampling is a standard operation for HMMs, although in this case the calculation burden can be reduced by careful inspection of the equations for the HMM forward algorithm [11]. By default, the state space of the model in Step 1 includes all of the known haplotypes in 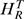 and the current-guess haplotypes in 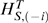. The computational burden of these calculations (both in terms of running time and memory usage) grows quadratically with the number of haplotypes and linearly with the number of SNPs. We later propose approximations to make these calculations more tractable on large datasets.

In Step 2, the algorithm uses each of the haplotypes in 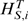 (which were sampled in Step 1) to impute new genotypes for SNPs in *U*. The HMM state space for this step includes only the reference panel haplotypes 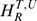. The imputation is accomplished by running the forward-backward algorithm for HMMs independently on each haplotype in 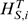 and then analytically determining the marginal posterior probabilities of the missing alleles – this process is simply a haploid analogue of the one used by IMPUTE v1. If we assume that both haplotypes were sampled from a population that conforms to Hardy-Weinberg Equilibrium (HWE), it is straightforward to convert these allelic probabilities to genotypic probabilities for individual *i*. Across iterations, we can then sum the posterior probabilities for each missing genotype as if they were weighted counts; at the end of a run, the final Monte Carlo posterior probabilities can be calculated by renormalizing these sums. By contrast with Step 1, the computational burden of these calculations grows only linearly with the number of haplotypes. Consequently, Step 2 can usually avoid the approximations needed to make Step 1 feasible, thereby allowing us to make full use of even very large reference panels.

By using both the reference panel and the study sample to inform phasing updates in Step 1, IMPUTE v2 uses more of the information in the data than most comparable methods [6],[11], which typically account for phase uncertainty using only the reference panel. At the same time, each iteration is relatively fast because untyped SNPs are imputed in a haploid framework rather than the more computationally intensive diploid framework that is used by other HMM methods. For example, one iteration of IMPUTE v2 will typically finish faster and use less computer memory than a run of IMPUTE v1 on the same dataset, although IMPUTE v2 tends to be slower than IMPUTE v1 on the whole since the new method requires multiple iterations. We explore the computational burden of the method in detail in the Results section.

### Scenario B

The structure of the dataset is more complex in this scenario than in the previous one, but we follow the same basic principles of imputation: phase the observed data, then impute alleles in each haplotype separately, conditioning on as much observed data as possible. Here, the goal of the phasing step is to end up with three sets of haplotypes: 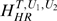, the known haploid reference panel haplotypes at SNPs in *T*, *U_1_*, and *U_2_*; 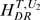, the unobserved diploid reference panel haplotypes at SNPs in *T* and *U_2_*; and 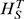, the set of unobserved study haplotypes at SNPs in *T*. If there are *N_DR_* individuals in the diploid reference panel, their haplotypes can be represented as 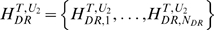, where 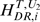 is the haplotype pair for diploid reference individual *i*.

The method begins by choosing initial guesses for the haplotypes in 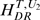 and 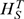 – as before, we choose haplotypes that are consistent with the observed genotype data but phased at random. Each MCMC iteration now includes five steps. First, we update every diploid reference individual *i*:

1. Sample a new haplotype pair 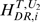 for individual *i* at SNPs in *T* and *U_2_*. This is accomplished by sampling from the conditional distribution 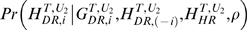.
2. Impute new alleles (in two independent haploid steps) for SNPs in *U_1_*, conditional on 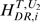, 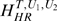, and *ρ*.
  In other words, we phase the observed data for diploid reference individual *i* by pooling information across both reference panels, then use these haplotypes in separate imputation steps based on the haploid reference panel. Up to this point, we have simply recapitulated Scenario A with different notation. Next, we update every study individual *i*:
3. Sample a new haplotype pair 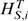 for individual *i* at SNPs in *T*. This is accomplished by sampling from the conditional distribution 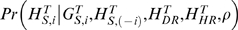.
4. Impute new alleles (in two independent haploid steps) for SNPs in *U_2_*, conditional on 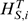, 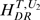, 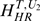, and *ρ*.
5. Impute new alleles (in two independent haploid steps) for SNPs in *U_1_*, conditional on 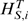, 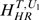, and *ρ*.

As is Scenario A, burn-in iterations are used only for phasing (Steps 1 and 3), while subsequent iterations cycle through all five steps.

In this algorithm, each study individual gains phasing information from all other individuals in the dataset, which can lead to very accurate haplotype estimates at typed SNPs when the total sample size is large. Once a study individual has sampled a new pair of haplotypes, the imputation step is broken into two parts: SNPs in *U_2_* are imputed using information from both the haploid and diploid reference panels (Step 4), and SNPs in *U_1_* are imputed using only the haploid reference panel (Step 5). This modeling choice highlights a core principle of our inference framework: we allow the method to naturally adapt to the amount of information in the data by conditioning only on observed genotypes, not imputed ones, at each step.

### Choice of conditioning states

As noted above, the HMM calculations underpinning our method require more running time and computer memory as more haplotypes are added to the state space of the model. This can be a problem for the phasing updates, whose computational burden increases quadratically with the number of haplotypes included in the calculation.

One solution, implemented in the phasing routine of the MACH software, is to use only a random subset of the available haplotypes for each update. For example, when sampling a new haplotype pair from 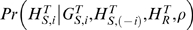 in Step 1 of our algorithm for Scenario A, we could use a random subset of *k* haplotypes drawn from 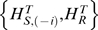 to build the conditional distribution, rather than the default approach of using all of the haplotypes. This approximation to the model will generally decrease accuracy, but it will also cause the computational burden of the phasing updates to increase linearly (for fixed *k*), rather than quadratically, with the number of chromosomes in the dataset.

We have developed another approximation that also constrains phasing updates to condition on a subset of *k* haplotypes. Rather than selecting haplotypes at random, our approach seeks to identify the *k* haplotypes that are in some sense “closest” to the haplotypes of the individual being updated. In genealogical terms, this amounts to focusing attention on the parts of the underlying tree where that individual's haplotypes are located. The idea is that haplotypes that reside nearby in the genealogical tree will the most informative about the haplotypes of interest.

The structure of the underlying genealogical tree is usually unknown (indeed, knowing the tree would essentially solve the phasing problem), so we frame the list of the *k* closest haplotypes as a random variable that gets updated for each individual at each MCMC iteration. To sample a new phase configuration for diploid individual *i*, we choose *k* conditioning states as follows: for each available non-self haplotype (including current-guess haplotypes for other diploid individuals), we calculate the Hamming distance to each of individual *i*'s current-guess haplotypes and store the minimum of these two distances. Then, we use the *k* haplotypes with the smallest distances to build the HMM and sample a new pair of haplotypes for individual *i*.

The transition and emission probabilities of our model [6] depend explicitly on *k*. The intuition is that, as *k* gets larger, jumps between different copied haplotypes should become less likely and those haplotypes should be copied with higher fidelity; this is because a chromosome will coalesce faster into a larger genealogy, leaving less time for recombination and mutation events to occur [18]. The underlying theory assumes that the haplotypes in question were sampled randomly from a population, which is clearly not the case when we select *k* haplotypes in the manner described above. To account for the fact that these haplotypes will find common ancestors (going backwards in genealogical time) more quickly than would *k* haplotypes chosen at random, we replace *k* with the *total number of available haplotypes* when specifying the HMM parameters for a phasing update.

We refer to this approximation as *informed* selection of conditioning states. While this method is built upon genealogical intuitions, we emphasize that no explicit genealogies are constructed in our inference scheme. One way of understanding our approach is by comparison to the phasing method of Kong et al. [19]. Their method uses rule-based techniques to phase putative “unrelateds” by identifying long stretches of identity-by-state (IBS) sharing between individuals, under the assumption that such sharing is caused by recent common descent. Our Hamming distance metric can be viewed as a way of identifying near-IBS sharing, and our method combines information across multiple closely related individuals in a model-based way rather than seeking perfect IBS matching between specific individuals. In this sense, our approximation can be viewed as a flexible middle ground between full conditional modeling (which uses all of the available haplotypes to phase an individual) and the Kong et al. method (which may use only a small fraction of the available haplotypes to phase an individual).

In our experience, imputation based on this informed method for choosing conditioning states is only trivially slower than otherwise identical analyses based on random state selection, effectively because the common HMM calculations take much longer than calculating all pairwise Hamming distances in the informed method. At the same time, the informed method can generally achieve the same phasing accuracy as the random method using many fewer states, or higher accuracy for a fixed number of states (data not shown). This is a major advantage because it is computationally expensive to add states to the model (i.e., to increase *k*). We therefore focus on the informed state selection method in this study, with the random method used only during MCMC burn-in, although both approaches are implemented in our software. We conduct an exploration of the parameter settings under informed selection, including the dependence of imputation accuracy on *k*, in Text S1, where we also discuss potential limitations of the informed state selection scheme.

### Modeling strategies for imputation datasets

In order to understand the modeling choices underlying our new imputation algorithm, it is crucial to consider the statistical issues that arise in imputation datasets. For simplicity, we will discuss these issues in the context of Scenario A, although we will also extend them to Scenario B in the Results section. Fundamentally, imputation is very similar to phasing, so it is no surprise that most imputation algorithms are based on population genetic models that were originally used in phasing methods. The most important distinction between phasing and imputation datasets is that the latter include large proportions of systematically missing genotypes.

Large amounts of missing data greatly increase the space of possible outcomes, and most phasing algorithms are not able to explore this space efficiently enough to be useful for inference in large studies. A standard way to overcome this problem with HMMs [6],[11] is to make the approximation that, conditional on the reference panel, each study individual's multilocus genotype is independent of the genotypes for the rest of the study sample. This transforms the inference problem into a separate imputation step for each study individual, with each step involving only a small proportion of missing data since the reference panel is assumed to be missing few, if any, genotypes.

In motivating our new imputation methodology, we pointed out that modeling the study individuals independently, rather than jointly, sacrifices phasing accuracy at typed SNPs; this led us to propose a hybrid approach that models the study haplotypes jointly at typed SNPs but independently at untyped SNPs. We made the latter choice partly to improve efficiency – it is fast to impute untyped alleles independently for different haplotypes, which allows us to use all of the information in large reference panels – but also because of the intuition that there is little to be gained from jointly modeling the study sample at untyped SNPs.

By contrast, the recently published BEAGLE [13] imputation approach fits a full joint model to all individuals at all SNPs. To overcome the difficulties caused by the large space of possible genotype configurations, BEAGLE initializes its model using a few ad-hoc burn-in iterations in which genotype imputation is driven primarily by the reference panel. The intuition is that this burn-in period will help the model reach a plausible part of parameter space, which can be used as a starting point for fitting a full joint model.

This alternative modeling strategy raises the question of whether, and to what extent, it is advantageous to model the study sample jointly at untyped SNPs. One argument [20] holds that there is no point in jointly modeling such SNPs because all of the linkage disequilibrium information needed to impute them is contained in the reference panel. A counterargument is that, as with any statistical missing data problem, the “correct” inference approach is to create a joint model of all observed and missing data. We have found that a full joint model may indeed improve accuracy on small, contrived imputation datasets (data not shown), and this leads us to believe that joint modeling could theoretically increase accuracy in more realistic datasets.

However, a more salient question is whether there is any *useful* information to be gained from jointly modeling untyped SNPs, and whether this information can be obtained with a reasonable amount of computational effort. Most imputation methods, including our new algorithm, implicitly assume that such information is not worth pursuing, whereas BEAGLE assumes that it is. We explore this question further in the sections that follow.

## Results

To test our new imputation method, we compared it with established methods on realistic datasets that fit the two scenarios described above.

### Scenario A

As an example of Scenario A, we used the 120 HapMap CEU parental haplotypes as a reference panel to impute genotypes in the WTCCC 1958 Birth Cohort (58 C) controls [1]. The 58 C samples were genotyped on the Affymetrix 500 K SNP chip, and the data were subjected to the SNP and sample filters specified in the WTCCC study [1]. Of the 1,502 58 C individuals, 1,407 were also genotyped on the Illumina 550 K chip, and 1,377 passed filtering in both datasets. We supplied only the latter set of individuals to the imputation methods, and we asked them to impute the 22,270 CEU HapMap SNPs on chromosome 10 that were represented on the Illumina chip but not the Affymetrix chip. We then used the imputed Illumina genotypes to evaluate the success of imputation based on the Affymetrix data.

#### Program settings

We used the following methods to perform the imputation: IMPUTE v1.0; MACH v0.1.10 with analytical (“mle”) imputation, where the model parameters were selected by running the “greedy” algorithm for 100 iterations on a random subset of 500 58 C samples, as suggested in the online tutorial that accompanies the software (http://www.sph.umich.edu/csg/abecasis/mach/tour/imputation.html); fastPHASE [11] v1.3.2 with 20 and 30 clusters (*K* = 20 and *K* = 30, in separate runs), 15 starts of the expectation-maximization (EM) algorithm to estimate model parameters, and 35 iterations per EM start (this version of fastPHASE automatically fits the clustering model to the reference panel and then imputes each study individual separately, conditional on the fitted model); BEAGLE v3.0.2 on default settings and with 50 iterations (rather than the default 10); and IMPUTE v2.0 with 40 and 80 conditioning states used for diploid updates at typed SNPs (*k* = 40 and *k* = 80, in separate runs) and 120 conditioning states (i.e., the full HapMap CEU panel) used for all haploid updates. We ran IMPUTE v2 with 10 burn-in iterations followed by 20 additional iterations. The first 3 burn-in iterations used random conditioning states for phasing updates, and all subsequent iterations used informed conditioning states. We discuss the motivations for these settings in Text S1.

We declined to include other imputation methods in this analysis because the Genetic Association Information Network (GAIN) Imputation Working Group is planning to publish a similar comparison using a broad cross-section of methods; our goal here is mainly to benchmark a new method. In order to speed up the analysis via our parallel computing facilities, we split chromosome 10 into 20 non-overlapping analysis chunks. Each imputation run spanned 7 Mb, with an additional 250 kb buffer on either side that was used for inference but omitted from the results – this buffer guards against a deterioration of imputation quality near the chunk edges. We ran every algorithm using the same analysis chunks.

#### Accuracy comparison

**Figure 3:**
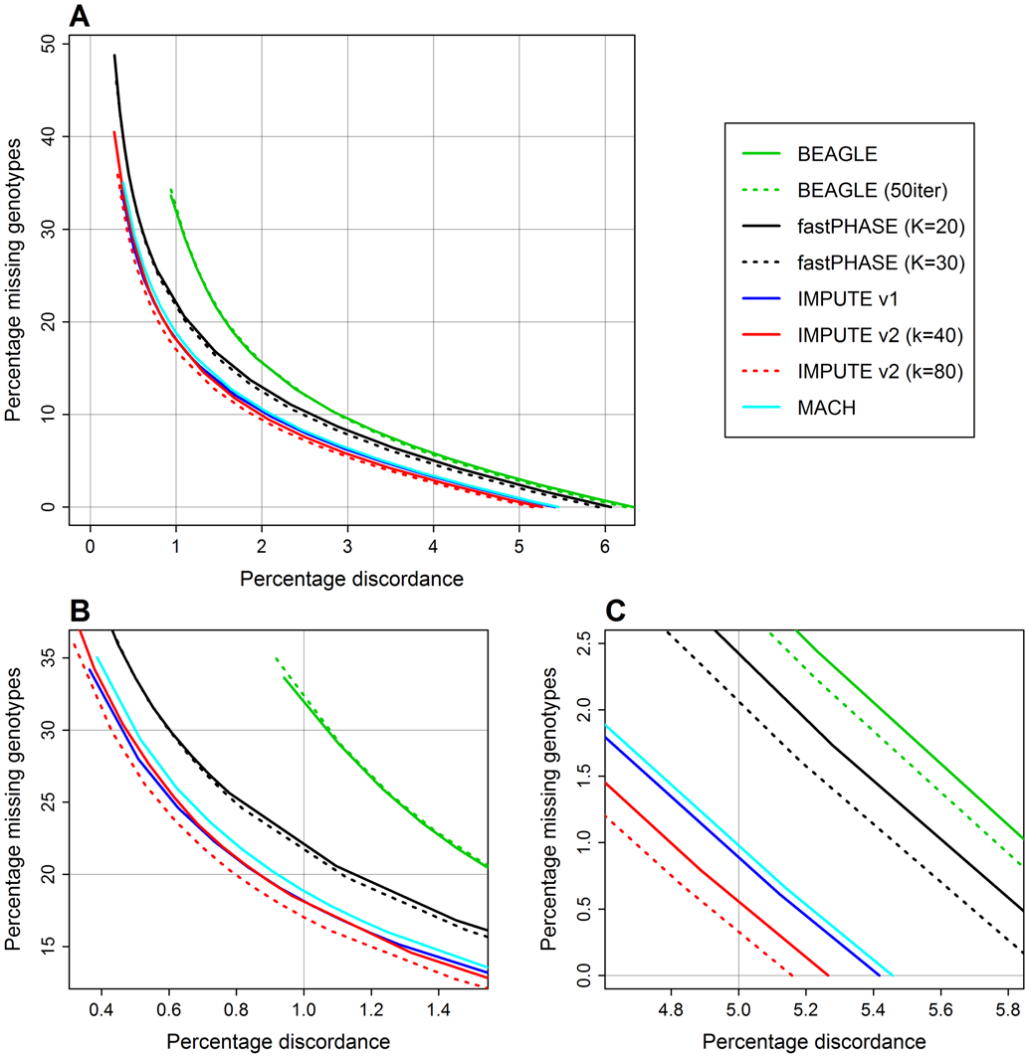
Percentage discordance versus percentage missing genotypes for Scenario A dataset. (A) Full range of results, corresponding to calling thresholds from 0.33 to 0.99. (B) Magnified results for calling thresholds near 0.99. (C) Magnified results for calling thresholds near 0.33.

The results of this analysis are shown in Figure 3. The *x*-axes in this figure display the discordance between imputed genotype calls and observed Illumina calls, which is a surrogate for imputation error rate; the *y*-axes display the percentage of genotypes for which no call was made. Each method's line is formed by considering several different calling thresholds for imputation posterior probabilities. For example, a certain number of maximum posterior probabilities will exceed a threshold of 0.9, and among these we can ask what percentage of the best-guess imputed genotypes disagree with the Illumina genotypes. This yields an *x*-coordinate, and the *y*-coordinate is simply the percentage of all imputed genotypes for which no posterior probability exceeds the threshold. We generated the lines on the plots by repeating these calculations for calling thresholds ranging from 0.33 to 0.99 for each method.

On these plots, lines that are below and to the left of other lines are more desirable. One interpretation is that, for a given level of missing data, an imputation method with a line further to the left has lower discordance with the external genotypes. Such plots allow us to evaluate competing methods in a more nuanced way than just looking at best-guess genotypes (which is equivalent to setting a single calling threshold of 1/3). We strongly emphasize, however, that the point of this exercise is not to determine an “optimal” calling threshold and use this to make hard calls of imputed genotypes for downstream analyses. Imputation results inherently contain more uncertainty than experimental genotype calls, and a host of methods have been developed to appropriately take this uncertainty into account when doing things like association testing [6]. Such methods are implemented in our freely available association testing software, SNPTEST.

Figure 3A shows the full results of this comparison. The curves are difficult to distinguish in this plot, so Figure 3B and 3C magnifies either end of the range to highlight the salient features. The grid lines in all three panels are shown at the same vertices to help convey the degree of magnification. The results can also be summarized by the best-guess error rate for each method (*x*-intercept on the plots): BEAGLE (default), 6.33%; BEAGLE (50 iterations), 6.24%; fastPHASE (*K* = 20), 6.07%; fastPHASE (*K* = 30), 5.92%; IMPUTE v1, 5.42%; IMPUTE v2 (*k* = 40), 5.23%; IMPUTE v2 (*k* = 80), 5.16%; MACH, 5.46%. Figure 3 shows that IMPUTE v1 (blue) achieved error rates that were consistently, if only slightly, lower than those of MACH (cyan) across the range of calling thresholds, and that both methods yielded lower error rates than fastPHASE (black) and BEAGLE (green). The IMPUTE v2 run with *k* = 40 (solid red line) attained similar accuracy to IMPUTE v1 at stringent calling thresholds (Figure 3B), although IMPUTE v2 gained a slight advantage at more lenient thresholds (Figure 3C). The IMPUTE v2 run with *k* = 80 (dotted red line) showed a small but consistent improvement over both IMPUTE v1 and the other IMPUTE v2 run.

#### Computational requirements

To describe the relative computational burdens of these methods, we re-ran each program on a more limited dataset on a single Linux server, which had four dual-core Intel Xeon processors (running at 2.33 GHz, with a 6.1 MB cache, and using a 64-bit architecture) and a total of 8 GB of RAM. Specifically, we repeated the analysis for the 4^th^, 8^th^, 12^th^, and 16^th^ chunks, each of which encompasses a 7.5 Mb region of chromosome 10 (centered, respectively, at positions 22.22 Mb, 50.22 Mb, 78.22 Mb, and 106.22 Mb in NCBI Build 35 coordinates). The average running times and memory requirements for these analyses are shown in Table 1.

**Table 1:** Running times and memory requirements for various algorithms in Scenario A.

Method	Avg. running time (min)	Avg. required RAM (MB)
BEAGLE	56	3100
BEAGLE (50iter)	392	3200
fastPHASE (K = 20)	397	8
fastPHASE (K = 30)	855	16
IMPUTE v1	43	1000
IMPUTE v2 (k = 40)	270	155
IMPUTE v2 (k = 80)	505	180
MACH	105	80

Running times are in minutes (min) and RAM requirements are in megabytes (MB). Each entry in the table is an average across four runs on different 7.5 Mb regions of chromosome 10. Each analysis included a reference panel of 120 chromosomes (CEU HapMap) and a study sample of 1,377 individuals genotyped on the Affymetrix 500 K SNP chip.

Table 1 shows that IMPUTE v1 was the fastest of the methods considered here, followed by BEAGLE (default), MACH, IMPUTE v2 (*k* = 40), BEAGLE (50 iterations), fastPHASE (*K* = 20), IMPUTE v2 (*k* = 80), and fastPHASE (*K* = 30). Conversely, fastPHASE required the least computer memory, followed by MACH, IMPUTE v2, IMPUTE v1, and BEAGLE. Note that, while IMPUTE v2 with *k* = 40 took about six times as long as IMPUTE v1, it needed less than 16% of the RAM; this is mainly a consequence of modeling SNPs in *U* as haploid in version 2, as opposed to diploid in version 1. We also note that both fastPHASE and MACH spent most of their running time fitting their models to the HapMap, and that both methods could probably decrease running times (via more lenient settings) without sacrificing much accuracy.

### Scenario B

We simulated Scenario B by modifying the WTCCC 58 C dataset as follows: First, we integrated the genotypes from the two SNP chips for the 1,377 shared 58 C individuals (see Text S1 for details), yielding a consensus set of 44,875 SNPs. Next, we split the 58 C samples into two groups: a diploid reference panel of 918 individuals (2/3 of the dataset) and a study sample of 459 individuals. To complete the reference panel, we added 120 haplotypes from the HapMap Phase II CEU data. We then created two Scenario B study sample datasets by masking the genotypes of SNPs unique to each chip in turn; there were 18,489 such SNPs on the Affymetrix chip and 22,219 such SNPs on the Illumina chip.

#### Modeling considerations

A full representation of Scenario B would include all HapMap SNPs that are polymorphic in the CEU panel. There were 138,592 such SNPs in our dataset, with 44,875 of these belonging to set *U_2_* and the remaining 93,717 to set *U_1_*. This data structure is problematic for most imputation methods because their modeling strategies are premised on a single reference panel in which most genotypes have been observed (i.e., some version of Scenario A). If the data from both reference panels in Scenario B were combined into a single panel, many reference SNPs (those in *U_1_*) would be missing large proportions of their genotypes, which could substantially decrease imputation accuracy in the study sample. Ad-hoc modifications of these approaches are not attractive either. For example, it would be possible for such methods to impute SNPs in *U_1_* in the diploid reference panel and then combine the observed and imputed genotypes to impute SNPs in *U_1_* and *U_2_* in the study sample, but failing to account for the uncertainty in the imputed reference genotypes would probably lead to overconfident and lower-quality inferences. Alternatively, it would be possible to perform separate imputation runs on the SNPs in {*U_1_*,*T*} and the SNPs in {*U_2_*,*T*}, but this approach is neither elegant nor convenient in a large association study.

To our knowledge, BEAGLE is the only method other than ours that has proposed a strategy for overcoming these difficulties. (This strategy is not discussed in the paper [13], but it is detailed in the documentation accompanying the BEAGLE v3.0 software.) When BEAGLE encounters multiple reference panels, as in Scenario B, it simply downweights the less complete panels during the burn-in stage of its model-fitting procedure. Specifically, every individual in the dataset is assigned a weight that reflects the completeness of that individual's genotypes – individuals with more missing data get lower weights, and therefore have less influence on the early steps of the model-fitting algorithm. This detail aside, BEAGLE still fits a joint model to the complete dataset in Scenario B, in contrast to the IMPUTE v2 approach of modeling the observed data jointly but the missing data independently.

In light of these considerations, we decided to create two versions of our Scenario B dataset: one that includes the *full* set of HapMap SNPs, and one in which the HapMap dataset is *restricted* to SNPs that were genotyped on at least one of the chips (i.e., in which all SNPs in *U_1_* have been removed). We used the latter dataset to broaden the range of methods that could be included in the comparison (at the cost of removing some of the complexity of Scenario B), and we used the former dataset to evaluate BEAGLE and IMPUTE v2 in a more realistic setting.

#### Program settings

In the restricted dataset, we used IMPUTE v1.0, IMPUTE v2.0, BEAGLE v3.0.2, fastPHASE v1.3.2, and PLINK [15] v1.03 to impute each chip's masked genotypes from the other chip's study sample genotypes and the reference panels. IMPUTE v2, BEAGLE, fastPHASE and PLINK used the 918 diploid individuals and the 120 HapMap CEU haplotypes as an expanded reference panel for imputation, while IMPUTE v1 was provided with only the HapMap reference panel. We ran IMPUTE v1, BEAGLE, and PLINK on their default imputation settings, and we also performed separate BEAGLE runs with 50 iterations (rather than the default 10). We ran fastPHASE with 20 and 30 clusters (*K* = 20 and *K* = 30, in separate runs), 15 starts of the EM algorithm to estimate model parameters, and 35 iterations per EM start. As in Scenario A, we first fit the fastPHASE clustering model to the reference data, then instructed the software to impute each of the 459 study individuals independently, conditional on the fitted model. Finally, we set IMPUTE v2 to use 40 and 80 conditioning states (*k* = 40 and *k* = 80, in separate runs) for phasing updates in both diploid panels and 1,956 reference panel states (2×918+120) for haploid imputation updates in the study sample. As before, we ran the algorithm for 30 MCMC iterations with the first 10 discarded as burn-in, and we specified that the algorithm should choose random conditioning states for phasing updates in the first 3 iterations and informed conditioning states thereafter.

On the full Scenario B dataset, we ran BEAGLE and IMPUTE v2 using the faster settings described above: 10 iterations for BEAGLE and *k* = 40 for IMPUTE v2. For each SNP that was not typed in the study sample, IMPUTE v2 used all observed reference panel chromosomes in each imputation step (1,956 states at SNPs in *U_2_* and 120 states at SNPs in *U_1_*).

#### Accuracy comparison on restricted dataset

**Figure 4:**
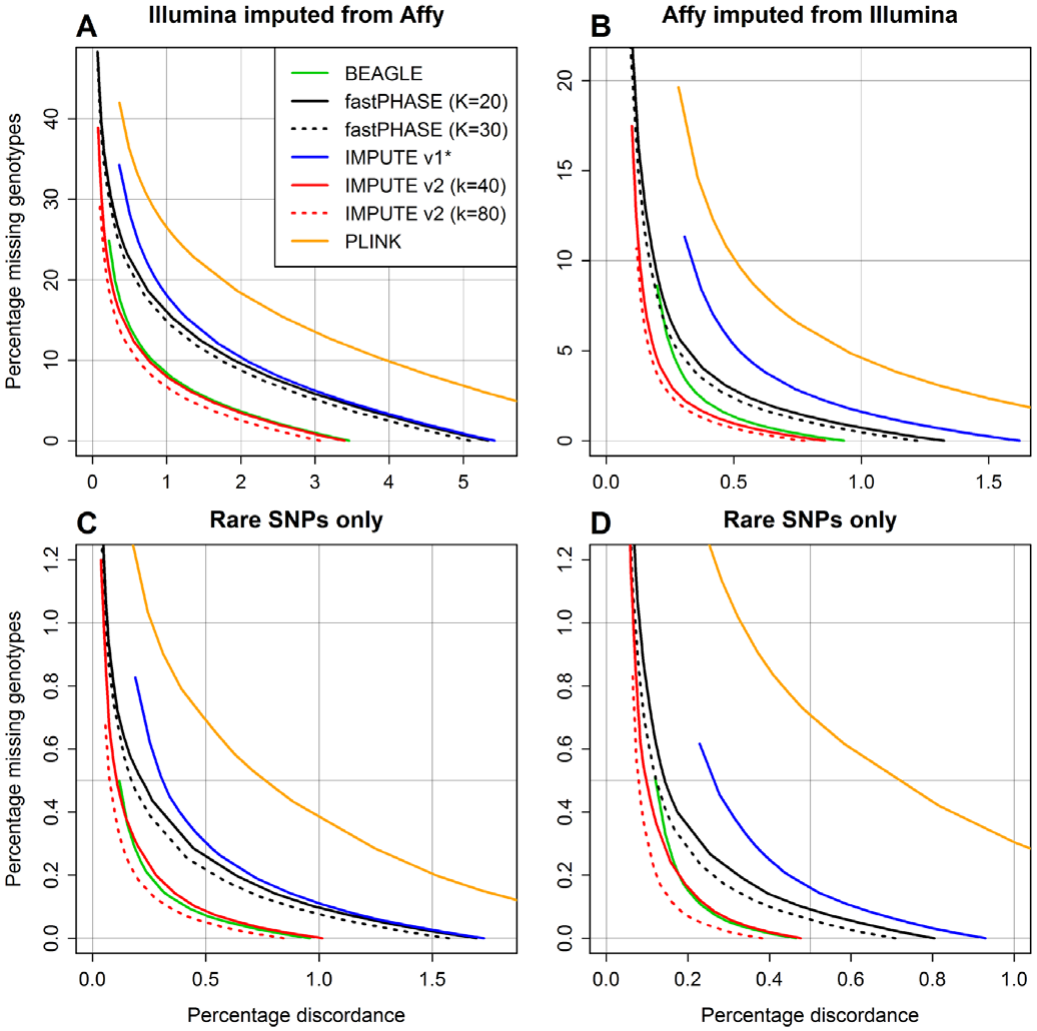
Percentage discordance versus percentage missing genotypes for restricted Scenario B dataset. (A) Results for masked Illumina genotypes imputed from Affymetrix genotypes in the study sample. (B) Results for masked Affymetrix genotypes imputed from Illumina genotypes in the study sample. (C) Results for masked Illumina genotypes (SNPs with MAF<5% only) imputed from Affymetrix genotypes in the study sample. (D) Results for masked Affymetrix genotypes (SNPs with MAF<5% only) imputed from Illumina genotypes in the study sample.

The results of our restricted Scenario B comparison are shown in Figure 4, using the same discordance vs. missing genotype percentage format as Figure 3. Note that each panel in this figure follows a different scale. The top two panels (Figure 4A and 4B) share a common set of grid lines, and the bottom two panels (Figure 4C and 4D) share a finer set of grid lines. We omitted the results from the BEAGLE run with 50 iterations since they were only trivially better than the results based on default settings.

Figure 4A shows the results for all Illumina-only SNPs imputed from Affymetrix genotypes, and Figure 4B shows the equivalent results for Affymetrix-only SNPs imputed from Illumina genotypes. One striking difference between these plots is that the imputations based on Illumina genotypes (Figure 4B) are generally more accurate. There are a number of possible explanations for this trend: the Illumina chip has a higher SNP density, and imputation generally improves as more SNPs are observed; the Affymetrix chip contains a larger proportion of rare SNPs, which are easier to impute on the whole, as we discuss below; and, while the Illumina SNPs were specifically chosen to predict, or “tag”, many of the common Affymetrix SNPs via the HapMap, the reverse is not true.

Regardless, one trend within Figure 4A and 4B is clear: with an expanded reference panel containing nearly 2,000 chromosomes, it is possible to improve imputation accuracy substantially over what is attainable with 120 chromosomes. For example, the IMPUTE v2 runs with *k* = 40 (solid red line) achieved best-guess discordance rates of 3.40% and 0.86% in Figure 4A and 4B, respectively, whereas the rates for IMPUTE v1 (which had access to only the HapMap reference panel; blue line) were 5.42% and 1.62%. BEAGLE (green), fastPHASE (black), and IMPUTE v2 (red) were all able to increase accuracy with the expanded reference panel, but the improvements for fastPHASE were smaller. BEAGLE (solid green line) and IMPUTE v2 with *k* = 40 (solid red line) yielded similar results: for BEAGLE, the best-guess discordance rates in Figure 4A and 4B were 3.46% and 0.93%. For IMPUTE v2, increasing the number of conditioning states used for phasing updates to *k* = 80 further reduced the discordance rates to 3.07% and 0.78%.

Unlike the other imputation methods with access to the expanded reference panel, PLINK achieved lower accuracy than IMPUTE v1; in Figure 4A and 4B, PLINK's best-guess discordances were 7.83% and 2.45%. We tried varying PLINK's settings from their defaults, including settings that were much more computationally rigorous, but these additional runs led to negligible improvements. PLINK is faster than the other methods considered here, which are all based on HMMs, but it also uses a simpler population genetics model. The multinomial haplotype frequency model that PLINK uses for imputation has fared poorly in recent comparisons of phasing methods [21]; its role in this analysis was to see if any accuracy is lost by using a simpler method to speed up imputation in a large and complex dataset.

Our results suggest that the model used by PLINK (which also underpins other imputation methods [7]) may be a liability in a dataset in which a large proportion of genotypes, including those in the reference panel, are unphased. However, we also note that PLINK's imputation functionality is still in beta testing. A recent study of Type 1 Diabetes [14] used a similar method to impute genotypes in a Scenario B dataset. Like PLINK, this method defines a multimarker tag for each SNP to be imputed, although in this case there is no phasing model since the tagging is based on correlations between unphased genotypes. It is not clear how this method would have fared in our comparison, but its similarities with PLINK imply that future studies might be better off using more sophisticated imputation methods.

Figure 4C and 4D mirrors Figure 4A and 4B, respectively, but these results are restricted to imputed SNPs with minor allele frequencies (MAFs) less than 5%—Figure 4C is based on 1,113 SNPs and Figure 4D is based on 1,979 SNPs. The same relative patterns remain, although the discordance and missing data percentages are lower because it is easier to guess most of the genotypes correctly and with high confidence at a rare SNP than a common one, simply because most genotypes at a rare SNP will be homozygous for the common allele. Among the most accurate methods, the best-guess discordances based on Affymetrix genotypes (Figure 4C) were 1.01% (IMPUTE v2, *k* = 40), 0.84% (IMPUTE v2, *k* = 80), and 0.97% (BEAGLE), as compared to 1.73% for HapMap-based imputation with IMPUTE v1; the discordances based on Illumina genotypes (Figure 4D) were 0.48% (IMPUTE v2, *k* = 40), 0.38% (IMPUTE v2, *k* = 80), and 0.46% (BEAGLE), as compared to 0.93% for IMPUTE v1.

#### Accuracy comparison on full dataset

**Figure 5:**
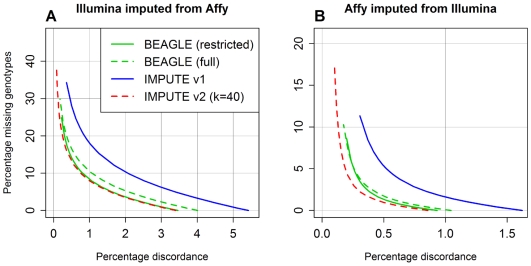
Percentage discordance versus percentage missing genotypes for full Scenario B dataset. (A) Results for masked Illumina genotypes imputed from Affymetrix genotypes in the study sample. (B) Results for masked Affymetrix genotypes imputed from Illumina genotypes in the study sample. Solid lines were obtained from the restricted Scenario B dataset (Figure 4) and are shown for reference; dashed lines were obtained from the full Scenario B dataset.

The results of our full Scenario B comparison are shown in Figure 5 in two panels that mirror Figure 4A and 4B. Although this dataset contains a large number of HapMap-only SNPs that were imputed here but not in the restricted dataset, we calculated discordance only at masked chip SNPs in the study sample, so the curves in Figure 4 and Figure 5 are based on exactly the same sets of masked genotypes. There are four curves in each panel: IMPUTE v2 in the full Scenario B dataset (*k* = 40; dashed red line); BEAGLE in the full dataset (default settings; dashed green line); BEAGLE in the restricted dataset (default settings; solid green line); and IMPUTE v1 in the restricted dataset (solid blue line). The first two curves (dashed lines) are the main focus of this comparison, and the latter two curves (solid lines) are carried over from Figure 4 for reference.

One important point about this figure is that the IMPUTE v2 curve is in exactly the same place as in Figure 4. It follows from our modeling approach that simply adding SNPs to the set *U_1_*, as we have done here, will not affect the imputation of SNPs in *U_2_*. Conversely, Figure 5 shows that adding SNPs to *U_1_* actually makes BEAGLE's imputation results worse at SNPs in *U_2_*: between the restricted and full datasets, the best-guess discordance increased from 3.46% to 4.01% in panel A and from 0.93% to 1.04% in panel B. We observed a similar decline in accuracy at rare SNPs, which are not shown separately in Figure 5. Hence, in the full Scenario B dataset, which we regard as a more realistic application of these methods, IMPUTE v2 achieves a best-guess discordance that is 15–18% smaller than BEAGLE's. In the Discussion, we propose an explanation for the change in BEAGLE's results between the full and restricted datasets.

A major goal of performing imputation in Scenario B (and extensions thereof) is to simultaneously use all available reference data in an integrated modeling framework. As such, it is also important to assess the quality of imputation at SNPs in *U_1_* (i.e., HapMap-only SNPs) in this context. To do so, we created a modified version of the full Scenario B dataset with observed Illumina genotypes in the study sample. We masked every 25^th^ Illumina SNP in both the study sample and the diploid reference panel, then ran BEAGLE and IMPUTE v2 as before. We repeated these steps for each of the 24 other possible sets of masked SNPs (i.e., after shifting the starting index), so that every Illumina SNP was masked and imputed exactly once.

Across these imputation runs, the best-guess discordance at masked SNPs in *U_1_* was 2.87% for IMPUTE v2 and 3.60% for BEAGLE – i.e., the discordance for IMPUTE v2 was 20% smaller than the discordance for BEAGLE.

#### Detecting minor allele copies at rare SNPs

While Figure 4 confirms that a large reference panel can improve imputation accuracy at rare and common SNPs alike, it is instructive to examine where the gains at rare SNPs are made. To evaluate this question, we took the results from Figure 4C and 4D and classified the kinds of errors made by each method's best-guess imputations (i.e., at a calling threshold of 1/3). We focused primarily on the ability of each method to detect copies of the minor allele. This is clearly an important quantity, but it is obscured by gross measures of accuracy, which are inherently dominated by homozygote-common genotypes at rare SNPs. We examined two classifications of erroneous minor allele calls: false positives (homozygous common called as heterozygous) and false negatives (heterozygous called as homozygous common). The results are shown in Table 2, where the false positive and false negative rates are expressed as percentages of the total number of homozygous common and heterozygous genotypes, respectively.

**Table 2:** False negative (FN) and false positive (FP) minor allele call rates at rare SNPs (MAF<5%) in Scenario B.

Method	Affymetrix 500 K data		Illumina 550 K data	
	FN calls (%)	FP calls (%)	FN calls (%)	FP calls (%)
BEAGLE	12.81	0.30	6.75	0.17
fastPHASE (K = 20)	21.12	0.28	11.07	0.15
fastPHASE (K = 30)	19.46	0.27	9.77	0.13
IMPUTE v1*	14.52	0.79	10.23	0.34
IMPUTE v2 (k = 40)	12.86	0.15	6.81	0.07
IMPUTE v2 (k = 80)	9.66	0.15	4.90	0.08
PLINK	32.79	0.53	25.63	0.40

The two columns on the left show results for Illumina-only genotypes imputed from Affymetrix 500 K data in the study sample, and the two columns on the right show results for Affymetrix-only genotypes imputed from Illumina 550 K data. The FN rates are expressed as percentages of genotypes that are truly heterozygous and the FP rates as percentages of genotypes that are truly homozygous common.

*: Unlike the other methods, IMPUTE v1 was not provided with the diploid reference panel. Consequently, these numbers are based on using a reference panel of 120 chromosomes to impute a study sample of 459 individuals.

Several insights emerge from this table. First, IMPUTE v2 was consistently among the best methods for reducing both false negatives and false positives, suggesting that our new approach is generally more accurate than others at imputing rare SNPs. Second, while most methods were much more likely to make false negative calls than false positive calls, IMPUTE v1 was relatively more inclined toward false positives. This outcome is consistent with the use of a smaller reference panel, which will tend to overestimate the population frequencies of rare alleles. At the same time, IMPUTE v1 missed fewer true heterozygote calls than did fastPHASE or PLINK, despite using a much smaller reference panel. This tendency for fastPHASE and PLINK to mistake rare heterozygotes as homozygous for the major allele is the main factor separating these methods from IMPUTE v2 and BEAGLE in Figure 4C and 4D. Conversely, BEAGLE and IMPUTE v2 with *k* = 40 showed similar false negative rates, but IMPUTE v2 made half as many false positive rare allele calls.

Putting these pieces together, it appears that IMPUTE v2 achieves higher accuracy than other methods at rare SNPs because it has both high sensitivity and high specificity for detecting rare alleles. These results show the strength of using a large reference panel for imputation, and of our particular approach to performing inference in that setting.

#### Computational requirements

As in Scenario A, we re-ran all programs on a single Linux server to assess their computational burdens in Scenario B. We used the same server and analysis chunks as before; the average running times and memory requirements across these four 7.5 Mb regions of chromosome 10 are shown in Table 3. (These numbers were averaged across both the masked Affymetrix and masked Illumina datasets, so eight runs contributed to each table entry.) Numbers in parentheses refer to the full Scenario B dataset, and all other numbers refer to the restricted dataset. Note that IMPUTE v1 was run on a version of the dataset that did not include the diploid reference panel.

**Table 3:** Running times and memory requirements for various algorithms in Scenario B.

Method	Avg. running time (min)	Avg. required RAM (MB)
BEAGLE	21 (70)	2500 (3200)
fastPHASE (K = 20)	530	12
fastPHASE (K = 30)	1100	20
IMPUTE v1*	5	260
IMPUTE v2 (k = 40)	409 (450)	80 (190)
IMPUTE v2 (k = 80)	790	120
PLINK	1.5	8

Running times are in minutes (min) and RAM requirements are in megabytes (MB). Each entry in the table is an average across eight runs, including four runs on different 7.5 Mb regions of chromosome 10 for study samples with either Affymetrix-only or Illumina-only SNPs masked. Each analysis included a haploid reference panel of 120 chromosomes (CEU HapMap), a diploid reference panel of 1836 chromosomes, and a study sample of 459 individuals. Numbers in parentheses represent analyses that included all SNPs that are polymorphic in the HapMap CEU panel; for the rest of the analyses, only SNPs that were genotyped on either the Affymetrix 500 K or Illumina 550 K chip were included in the HapMap dataset.

*: Unlike the other methods, IMPUTE v1 was not provided with the diploid reference panel. Consequently, these numbers are based on using a reference panel of 120 chromosomes to impute a study sample of 459 individuals.

PLINK was the fastest of these methods, followed by IMPUTE v1, BEAGLE, IMPUTE v2, and fastPHASE. PLINK also required the least RAM, followed by fastPHASE, IMPUTE v2, IMPUTE v1, and BEAGLE. While BEAGLE was quite fast, it also required more than ten times as much RAM as any other method (at least 2.5 GB per 7.5 Mb region of the genome). BEAGLE includes an option to decrease memory usage, but this would come at the cost of increased running time.

We emphasize that IMPUTE v1 was among the fastest methods in this comparison only because it was assigned a much smaller problem: its reference panel contained 120 phased haplotypes, while every other method confronted a panel with 1,956 chromosomes, most of which were unphased. Using the knowledge that IMPUTE v1's computational burden grows quadratically with the number of chromosomes in the reference panel, we can project that it would have required over 1,300 minutes and 69,000 MB of RAM to run the program on a single 7.5 Mb analysis chunk with a reference panel of that size (which would also have needed to be phased ahead of time). This highlights a major advantage of our new modeling strategy: whereas IMPUTE v1 becomes computationally intractable as the reference panel grows, IMPUTE v2 remains competitive (both in computational burden and imputation accuracy) while allowing more flexibility (such as multiple, unphased, and/or incomplete reference panels).

Another advantage of our approach can be seen by comparing the running times of the restricted and full datasets for BEAGLE and IMPUTE v2. The average BEAGLE run took 3.3 times longer in the full dataset than in the restricted dataset, whereas the IMPUTE v2 running time increased by factor of just 1.1. For comparison, the total number of SNPs in the dataset increased by a factor of 3.1. This contrast between the methods arises from the way they model SNPs in *U_1_*: IMPUTE v2 models only the reference panel at such SNPs, whereas BEAGLE tries to model all individuals in the dataset. We regard the full dataset as a more realistic application of these methods, so we believe that the parenthetical running times in Table 2 offer the best comparison between BEAGLE and IMPUTE v2.

## Discussion

In this study we introduced a new method for genotype imputation in large association studies. Our method, IMPUTE version 2, follows a flexible inference framework that uses more of the information in the data than many comparable methods, thereby improving accuracy, while remaining computationally tractable on large datasets. This approach is well-suited to the kinds of datasets that will become available in next-generation association studies: it can handle large reference panels, including ones with unphased and incomplete genotypes, and it can also integrate multiple reference panels containing different sets of SNPs.

### Scenario A

The observation that IMPUTE v2 can achieve lower error rates than IMPUTE v1 in Scenario A validates our new approach. At the same time, the absolute improvement is small, as can be seen in Figure 3 by comparing the separation between IMPUTE v1 and v2 with the separation between IMPUTE v1 and MACH, which typically yield very similar results in our experience. We have also performed separate experiments in which IMPUTE v2 achieves much higher phasing accuracy than IMPUTE v1 at SNPs in *T*, but where the improvements in HapMap-based imputation of SNPs in *U* remain modest (data not shown). We suggest that this disconnect between phasing accuracy and imputation accuracy is caused by the inherent limitations of a small reference panel; in other words, we posit that existing models would not attain substantially lower imputation error rates with the current HapMap panel *even if we knew the phase of the study genotypes perfectly*.

In the wake of these results, we suspect that the accuracy improvement of IMPUTE v2 over IMPUTE v1 is not practically meaningful for imputation based on the HapMap Phase II data. However, given that IMPUTE v1's computational requirements scale quadratically with the number of chromosomes in the reference panel while IMPUTE v2's requirements grow linearly, the newer version may become more computationally favorable as baseline reference panels grow in the future. For example, expanding the HapMap reference panel in this study to 800 chromosomes (which is roughly the size anticipated for each panel in the 1,000 Genomes Project) would lead to similar running times for both versions of IMPUTE, but version 2 would need only 2% of the computer memory required by version 1. At the same time, IMPUTE v2 would probably achieve higher accuracy, and its computational advantages over IMPUTE v1 would continue to grow with larger reference panels.

### Scenario B

In our Scenario B dataset, we demonstrated that an expanded reference panel containing thousands of chromosomes can greatly improve accuracy over what is possible based on the HapMap alone, although these gains are limited to the subset of HapMap SNPs that are included on multiple genotyping chips. This finding is consistent with the conclusions of the recent BEAGLE paper [13]. IMPUTE v2 was consistently among the most accurate methods we considered. For example, IMPUTE v2 attained best-guess error rates that were 15–20% lower than those of its closest competitor (BEAGLE) in a realistic representation of Scenario B.

Rare SNPs are of particular interest because of an increasing awareness that such SNPs may underlie common, complex diseases, and because imputation methods gain the most power over tagging approaches at such SNPs [6],[11]. Expanded reference panels ought to allow rare SNPs to be imputed much more accurately than they can be with the HapMap panel, and our method is able to exploit this information more effectively than competing methods. Relative to IMPUTE v1 (which had access to only the HapMap reference panel) and BEAGLE, the main improvement of IMPUTE v2 is to increase specificity by cutting down on false positive heterozygous calls; relative to fastPHASE and PLINK, the main improvement is to increase sensitivity by cutting down on false negative heterozygous calls.

### Modeling issues in imputation datasets

Throughout this study we have touched on the fundamental modeling difficulties that arise in imputation datasets, and we have discussed various strategies that have been proposed to solve these problems. In particular, we have contrasted the BEAGLE approach of full joint modeling with the IMPUTE v2 approach, which phases the observed data jointly but imputes the missing alleles in different haplotypes independently.

Based on the results seen here and elsewhere [13], we claim that BEAGLE gains very little useful information through joint modeling of entire imputation datasets. Consider these lines of evidence:

1. In our Scenario B comparison, BEAGLE's accuracy at SNPs in *U_2_* actually decreased when SNPs were added to *U_1_*. This is highly counterintuitive: it is hard to explain why adding HapMap-only SNPs to a dataset, without changing any of the data in the rest of a region, should have a noticeable effect on the imputation of SNPs in an expanded reference panel, let alone a negative effect.
2. Browning and Browning (2009) observed that BEAGLE attained better accuracy by subdividing a study sample and fitting the model separately to each subsample (along with the complete reference panel) than by simply fitting the model to the entire dataset – indeed, this subdividing strategy is now recommended as standard practice by the authors. The benefits of subdividing the sample were attributed to “model averaging”, but that is not an apt description of the process since each individual in the dataset is subjected to only a single model fit. Some model fits are probably better than others due to the stochastic nature of the algorithm, but some are also worse, so there is no reason to expect systematic improvements from this strategy if the model is working properly.
3. Browning and Browning (2009) also observed that, for a fixed study sample of 188 individuals, BEAGLE's accuracy consistently improved relative to that of IMPUTE v1 as the size of the reference panel increased. No mechanistic rationale was provided to explain this trend.

The first two points document strange behavior of the BEAGLE method: apparently, adding data – whether in the form of additional SNPs or additional individuals in the study sample – can cause BEAGLE's imputation accuracy to decrease. More specifically, it seems that *increasing the proportion of missing data* harms BEAGLE's inferences. This suggests an explanation for the third point above: as the reference panel grew and the study sample remained fixed, the total proportion of missing genotypes in the sample decreased, thereby generating datasets that were relatively less harmful to BEAGLE.

In our view, these disparate observations point to a single underlying cause: joint modeling of untyped SNPs is generally ineffective, and it grows progressively worse as the space of missing genotypes expands. BEAGLE was competitive in our analyses, so its modeling strategy may have some merit, but it is also possible that BEAGLE's success came in spite of the joint modeling framework, not because of it. A better alternative might be to embed the same clustering model in a framework like the ones used by fastPHASE or IMPUTE v2. We suggest that further scrutiny be applied before a full joint model is used in general applications. Comparisons like ours, and others [13], are necessarily restricted to artificially small datasets, but we have shown that these “toy” datasets can mask problems that might occur in more realistic settings, which will often include larger amounts of missing data. In practice, the accuracy levels and running times achieved by BEAGLE in our study may represent best-case scenarios rather than standard results.

These considerations apply to imputation datasets in general, but it is particularly interesting to examine them in the context of multiple reference panels genotyped on different sets of SNPs. BEAGLE's joint approach to such datasets is flexible, but we have seen that it can lose accuracy when certain kinds of new data are added. Conversely, IMPUTE v2's multi-panel modeling strategy responds intuitively to new sources of information like additional individuals or SNPs. This property makes it easy to predict how IMPUTE v2 will perform in larger and more complex datasets than the ones used here, whereas the same cannot necessarily be said for BEAGLE.

More broadly, we believe that any imputation algorithm should strive to incorporate as much of the available reference information as possible while remaining easy to use. For example, in Scenario B it is desirable to simultaneously impute the SNPs in the expanded panel (to improve accuracy) and the SNPs represented only in the HapMap (to maintain genomic coverage). IMPUTE v2 provides an integrated framework for handling this kind of problem: it is flexible enough to handle numerous variations of Scenarios A and B, yet it remains tractable by focusing computational effort on the parts of the dataset that are most informative.

### Extensions

The expanded reference panel we considered was constituted by controls genotyped on multiple SNP chips, but other kinds of new reference panels will also become available in the near future. For example, the HapMap Project has recently augmented its Phase II data with additional samples from both the original HapMap locations and new locations aimed at capturing more human genetic diversity. These samples have all been genotyped on multiple, largely non-overlapping SNP chips, and could be used for imputation in the same way as the controls in our Scenario B. In addition, the 1,000 Genomes project is currently pursuing whole-genome sequencing of hundreds of individuals sampled from broad geographic regions in Africa, East Asia, and Europe. One aim of the project is to generate high-quality haplotypes for these individuals, including near-complete coverage of SNPs with population MAFs of 1% or more. This resource will increase the utility of imputation approaches by expanding both the number of chromosomes in the reference set and the number of SNPs that can be imputed.

Our method is well-suited to this kind of dataset: in addition to its ability to accurately impute rare SNPs, which will constitute most of the new variants in the 1,000 Genomes data, IMPUTE v2 expends relatively little computational effort on haploid imputation steps. This means that, for a given SNP chip typed in a given study sample, doubling the number of untyped variants in a phased reference panel will increase the computational burden of imputation by a factor of less than two. By contrast, other imputation methods (such as IMPUTE v1, BEAGLE, and fastPHASE) would slow down by a factor of at least two.

One major use of our new method (and of imputation methods generally) will be to facilitate meta-analyses [9],[10], which combine samples from studies of similar diseases to increase the chances of detecting low-penetrance risk alleles. For this application, we might expect to repeat Scenario B for a number of different study samples genotyped on different SNP chips. Rather than re-phase the diploid reference panel for each study sample, we can save time by simply storing the posterior samples from a single run of phasing the reference panel, then read these sampled haplotypes from memory when processing each study sample. This functionality is implemented in our software.

IMPUTE v2 is already fast enough to use in large association studies, but we also have plans to make it faster. We believe that the software can gain some speed simply by optimizing the code, but we also have plans to implement an analytical speed-up for the HMM forward-backward calculations [22] that may further decrease running times by a factor of five or so.

Finally, while we described our imputation approach in terms of two specific scenarios involving the HapMap, it could in fact be generalized to include any number of reference panels of any type (phased/unphased, complete/incomplete) so long as their SNP sets follow a hierarchy such as the ones laid out in Figure 1 and Figure 2. We envision that IMPUTE v2 will be used in a variety of situations. For example, it may soon become standard practice to combine the HapMap Phase II and Phase III datasets to create a compound reference panel like the one in Scenario B, except with all of the reference data phased. Another plausible situation is the version of Scenario B that we described, in which a large set of controls is used to impute genotypes in cases; we discuss some concerns about association testing in this setting in Text S1 and Figure S2. IMPUTE v2 will also be applied in populations beyond the UK controls used in this study, and we expect that its performance will follow trends much like those observed for similar imputation methods [23],[24].

Our modeling strategy is flexible and fast, and it is general enough that it could be adopted by other imputation methods. We believe that this intuitive way of thinking about imputation datasets will benefit next-generation association studies, and that IMPUTE v2 will prove to be a useful tool for finding subtle signals of association.

## Supporting Information

Figure S1 Percentage discordance between best-guess imputed and observed Illumina genotypes for various parameter settings of IMPUTE v2. These results were obtained from a 2 Mb region of chromosome 10 in the Scenario B dataset.
(0.27 MB TIF)

Figure S2 Expected versus observed p-values for additive association tests between the 58 C and UKBS control groups, where the UKBS genotypes have been imputed from 58 C genotypes. (A) p-p plot for common (MAF≥5%) SNPs. (B) p-p plot for rare SNPs. The 95% concentration band is shown in grey, and the y = x line is shown in red.
(0.13 MB TIF)

Table S1 Convergence statistics for various parameter settings of IMPUTE v2. For each combination of burn-in and main iterations, the number shown is the percentage of imputed genotypes for which the R convergence statistic was greater than 1.02 across 10 independent runs of the algorithm. The results are stratified into genotypes at 100 common SNPs (left) and genotypes at 24 rare SNPs (right); for rare SNPs, only genotypes that include the minor allele were used in the calculations. These results were obtained from a 2 Mb region of chromosome 10 in our Scenario B dataset, using IMPUTE v2 with *k* = 30 (results with *k* = 100 were similar).
(0.03 MB PDF)

Text S1 Performance of IMPUTE v2 under various parameter settings; Convergence of IMPUTE v2 algorithm; Limits of informed conditioning approximation; Integrating genotypes from two SNP chips; Association testing of cases imputed from controls.
(0.28 MB PDF)

## References

[1] The Wellcome Trust Case Control Consortium (2007). Genome-wide association study of 14,000 cases of seven common diseases and 3,000 shared controls.. Nature.

[2] Rioux JD, Xavier RJ, Taylor KD, Silverberg MS, Goyette P (2007). Genome-wide association study identifies new susceptibility loci for Crohn disease and implicates autophagy in disease pathogenesis.. Nat Genet.

[3] Gudmundsson J, Sulem P, Manolescu A, Amundadottir LT, Gudbjartsson D (2007). Genome-wide association study identifies a second prostate cancer susceptibility variant at 8q24.. Nat Genet.

[4] Frazer KA, Ballinger DG, Cox DR, Hinds DA, Stuve LL (2007). A second generation human haplotype map of over 3.1 million SNPs.. Nature.

[5] Servin B, Stephens M (2007). Imputation-based analysis of association studies: candidate regions and quantitative traits.. PLoS Genet.

[6] Marchini J, Howie B, Myers S, McVean G, Donnelly P (2007). A new multipoint method for genome-wide association studies by imputation of genotypes.. Nat Genet.

[7] Lin DY, Hu Y, Huang BE (2008). Simple and efficient analysis of disease association with missing genotype data.. Am J Hum Genet.

[8] Nicolae DL (2006). Testing untyped alleles (TUNA)-applications to genome-wide association studies.. Genet Epidemiol.

[9] Zeggini E, Scott LJ, Saxena R, Voight BF, Marchini JL (2008). Meta-analysis of genome-wide association data and large-scale replication identifies additional susceptibility loci for type 2 diabetes.. Nat Genet.

[10] Barrett JC, Hansoul S, Nicolae DL, Cho JH, Duerr RH (2008). Genome-wide association defines more than 30 distinct susceptibility loci for Crohn's disease.. Nat Genet.

[11] Scheet P, Stephens M (2006). A fast and flexible statistical model for large-scale population genotype data: applications to inferring missing genotypes and haplotypic phase.. Am J Hum Genet.

[12] Browning SR, Browning BL (2007). Rapid and accurate haplotype phasing and missing-data inference for whole-genome association studies by use of localized haplotype clustering.. Am J Hum Genet.

[13] Browning BL, Browning SR (2009). A unified approach to genotype imputation and haplotype-phase inference for large data sets of trios and unrelated individuals.. Am J Hum Genet.

[14] Barrett JC, Clayton DG, Concannon P, Akolkar B, Cooper JD (2009). Genome-wide association study and meta-analysis find that over 40 loci affect risk of type 1 diabetes.. Nat Genet.

[15] Purcell S, Neale B, Todd-Brown K, Thomas L, Ferreira MA (2007). PLINK: a tool set for whole-genome association and population-based linkage analyses.. Am J Hum Genet.

[16] Li N, Stephens M (2003). Modeling linkage disequilibrium and identifying recombination hotspots using single-nucleotide polymorphism data.. Genetics.

[17] Myers S, Bottolo L, Freeman C, McVean G, Donnelly P (2005). A fine-scale map of recombination rates and hotspots across the human genome.. Science.

[18] Wakeley J (2007). Coalescent Theory : An Introduction.

[19] Kong A, Masson G, Frigge ML, Gylfason A, Zusmanovich P (2008). Detection of sharing by descent, long-range phasing and haplotype imputation.. Nat Genet.

[20] Guan Y, Stephens M (2008). Practical issues in imputation-based association mapping.. PLoS Genet.

[21] Marchini J, Cutler D, Patterson N, Stephens M, Eskin E (2006). A comparison of phasing algorithms for trios and unrelated individuals.. Am J Hum Genet.

[22] Chen W, Li Y, Abecasis G (2008). State Space Reduction in Hidden Markov Model for Haplotyping, Imputation and Analysis of Shotgun Sequence Data.

[23] Zhao Z, Timofeev N, Hartley SW, Chui DH, Fucharoen S (2008). Imputation of missing genotypes: an empirical evaluation of IMPUTE.. BMC Genet.

[24] Huang L, Li Y, Singleton AB, Hardy JA, Abecasis G (2009). Genotype-imputation accuracy across worldwide human populations.. Am J Hum Genet.

