# Supplementary figures and images for: A Flexible and Accurate Genotype Imputation Method for the Next Generation of Genome-Wide Association Studies
Source: PLoS Genet. 2009 Jun 19;5(6):e1000529. doi: 10.1371/journal.pgen.1000529 (PMC2689936; doi:10.1371/journal.pgen.1000529)

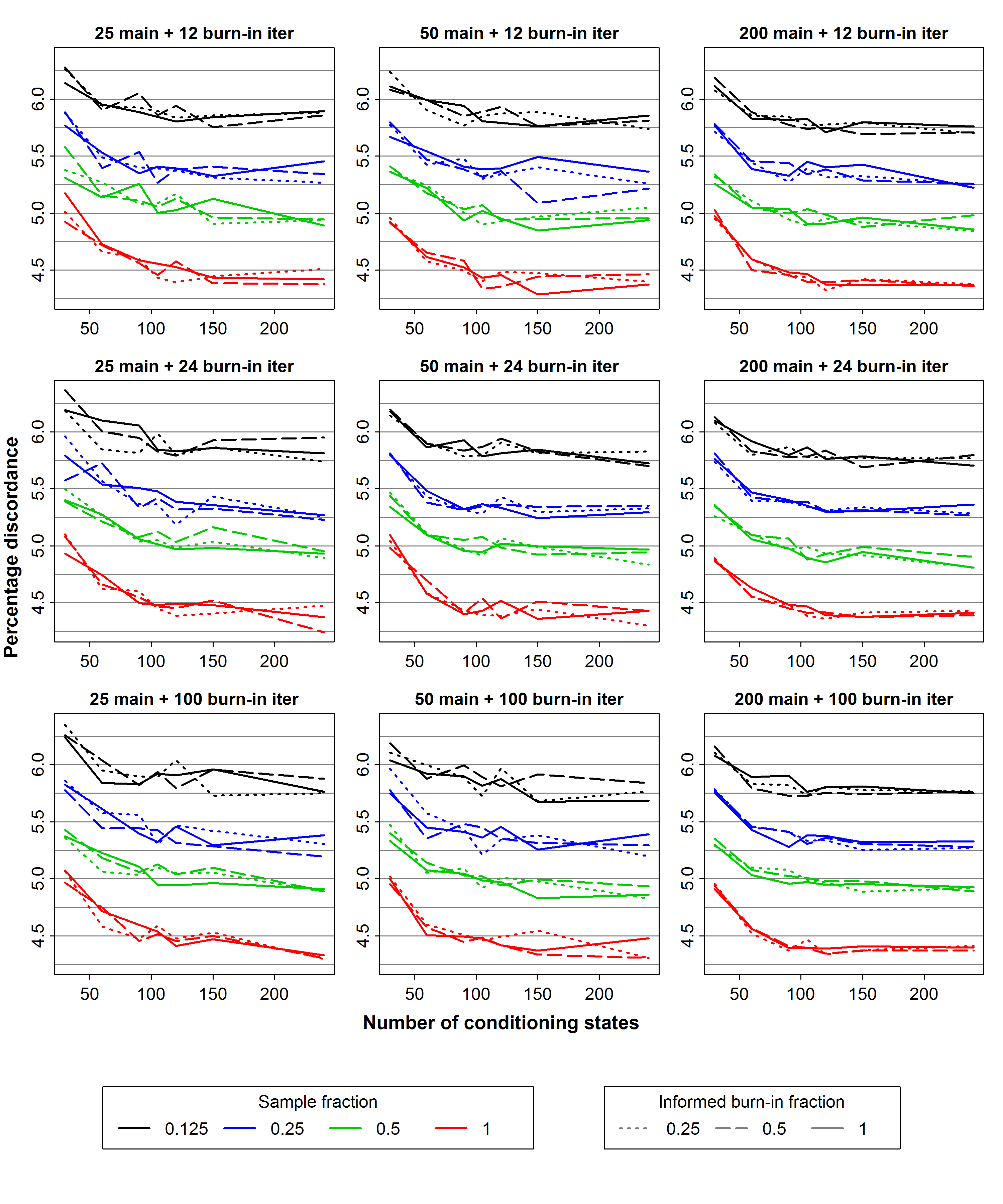

Supplement: Figure S1 — Percentage discordance between best-guess imputed and observed Illumina genotypes for various parameter settings of IMPUTE v2. These results were obtained from a 2 Mb region of chromosome 10 in the Scenario B dataset. (0.27 MB TIF) [file pgen.1000529.s001.tif]

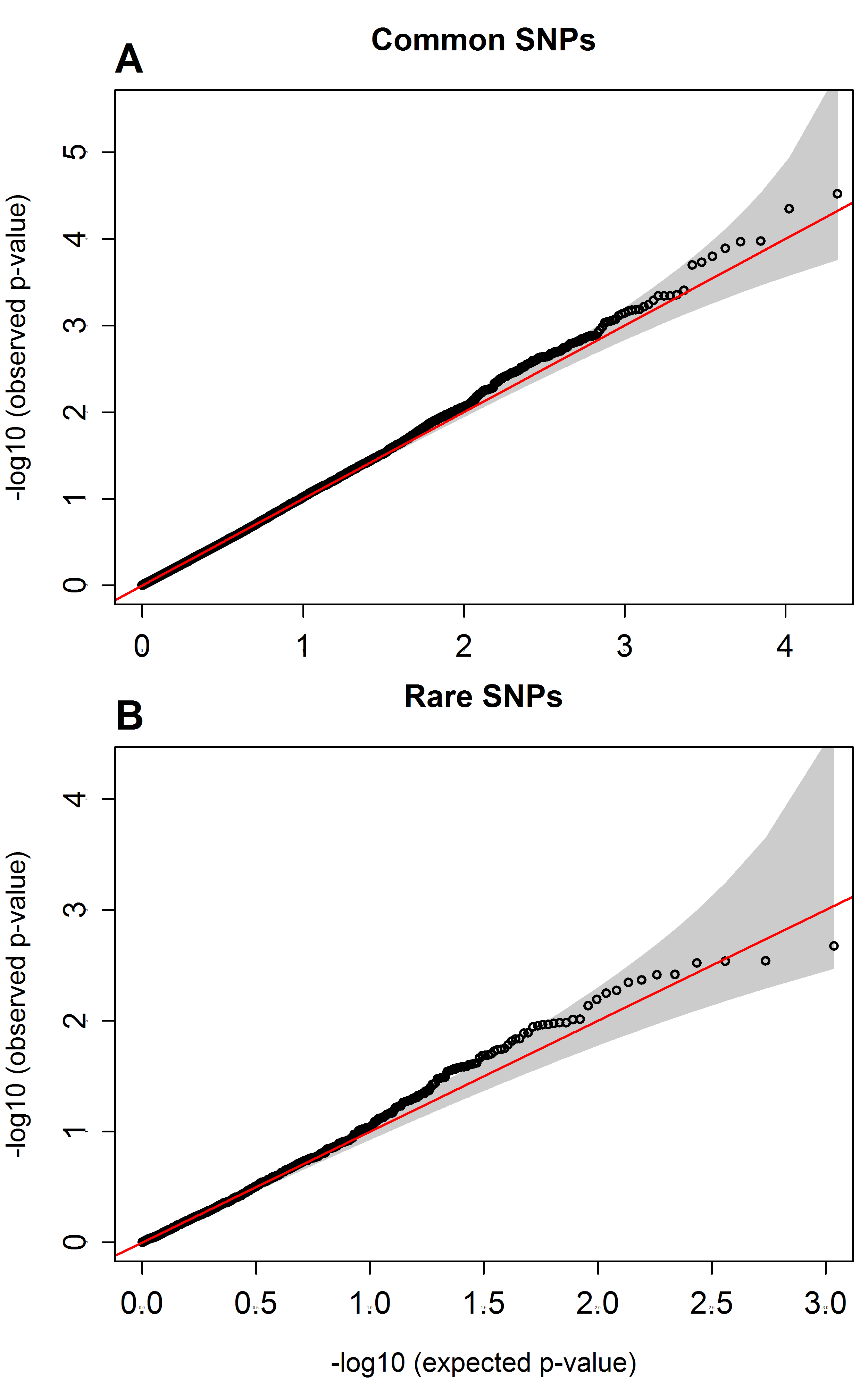

Supplement: Figure S2 — Expected versus observed p-values for additive association tests between the 58 C and UKBS control groups, where the UKBS genotypes have been imputed from 58 C genotypes. (A) p-p plot for common (MAF≥5%) SNPs. (B) p-p plot for rare SNPs. The 95% concentration band is shown in grey, and the y = x line is shown in red. (0.13 MB TIF) [file pgen.1000529.s002.tif]
