# Supplementary material for: A Flexible and Accurate Genotype Imputation Method for the Next Generation of Genome-Wide Association Studies
Source: PLoS Genet. 2009 Jun 19;5(6):e1000529. doi: 10.1371/journal.pgen.1000529 (PMC2689936; doi:10.1371/journal.pgen.1000529)
Supplement: Table S1 — Convergence statistics for various parameter settings of IMPUTE v2. For each combination of burn-in and main iterations, the number shown is the percentage of imputed genotypes for which the R convergence statistic was greater than 1.02 across 10 independent runs of the algorithm. The results are stratified into genotypes at 100 common SNPs (left) and genotypes at 24 rare SNPs (right); for rare SNPs, only genotypes that include the minor allele were used in the calculations. These results were obtained from a 2 Mb region of chromosome 10 in our Scenario B dataset, using IMPUTE v2 with k = 30 (results with k = 100 were similar). (0.03 MB PDF) [file pgen.1000529.s003.pdf]

Table S1

<table>
<tr><th colspan="2" rowspan="2">Common SNPs</th><th colspan="3">Main iterations</th><th rowspan="5"></th><th colspan="2" rowspan="2">Rare SNPs</th><th colspan="3">Main iterations</th></tr>
<tr><th>20</th><th>50</th><th>100</th><th>20</th><th>50</th><th>100</th></tr>
<tr><th rowspan="3">Burn-in iterations</th><th>10</th><td>4.68</td><td>1.12</td><td>0.17</td><th rowspan="3">Burn-in iterations</th><th>10</th><td>3.14</td><td>0.14</td><td>0</td></tr>
<tr><th>25</th><td>4.85</td><td>1.01</td><td>0.13</td><th>25</th><td>3.14</td><td>0.41</td><td>0.27</td></tr>
<tr><th>100</th><td>4.52</td><td>0.87</td><td>0.08</td><th>100</th><td>2.82</td><td>0.68</td><td>0</td></tr>
</table>
