# Supplementary material for: A Flexible and Accurate Genotype Imputation Method for the Next Generation of Genome-Wide Association Studies
Source: PLoS Genet. 2009 Jun 19;5(6):e1000529. doi: 10.1371/journal.pgen.1000529 (PMC2689936; doi:10.1371/journal.pgen.1000529)
Supplement: Text S1 — Performance of IMPUTE v2 under various parameter settings; Convergence of IMPUTE v2 algorithm; Limits of informed conditioning approximation; Integrating genotypes from two SNP chips; Association testing of cases imputed from controls. (0.28 MB PDF) [file pgen.1000529.s004.pdf]

A Flexible and Accurate Genotype Imputation Method for the Next Generation of Genome-Wide Association Studies

SUPPLEMENTARY MATERIAL

Bryan N. Howie, Peter Donnelly, and Jonathan Marchini

Performance of IMPUTE v2 under various parameter settings

To better understand how the results of IMPUTE v2 depend on its parameter settings, we selected a 2 Mb region (chromosome 10 positions 79-81 Mb in NCBI Build 35 coordinates) from our Scenario B dataset. We instructed the method to impute the 359 SNPs in this region that are typed only on the Illumina chip, using information from the expanded reference panel and from 469 Affymetrix SNPs whose genotypes were observed in the study sample (the latter SNP set includes a 250 kb buffer on either side of the region to protect against edge effects). We repeated the analysis under all possible combinations of the following settings: 12, 24, and 100 burn-in iterations; 25, 50, and 200 main iterations; and 30, 60, 90, 105, 120, 150, and 240 conditioning states (k) per phasing update. We also wanted to assess the effects of sample size, so we took nested subsamples containing 12.5%, 25%, and 50% of the diploid reference panel and the study sample.

Another question we have considered is whether to choose informed conditioning states for all iterations or to devote some of the initial burn-in iterations to the random conditioning approach. The intuition is that the informed method might get stuck in a local mode of the posterior distribution if the initial random phasing yielded an unfavorable configuration. To address this, we allocated different numbers of informed conditioning iterations during the burn-in period. Each run either used the informed method for all burn-in iterations or just the last 25% or 50% of burn-in iterations.

The results of this experiment are shown in Figure S1. Each row of plots corresponds to a fixed number of burn-in iterations, and each column corresponds to a fixed number of main iterations. Subsampled datasets are represented in different colors;

to enhance comparability across subsamples, all results in this figure were calculated using only the 12.5% of study individuals that were common to all four datasets. Runs with different fractions of their burn-in devoted to the informed conditioning method are drawn using dotted, dashed, and solid lines (for 25%, 50%, and 100% of burn-in iterations based on informed conditioning, respectively). The *x*-axis of each plot tracks the number of conditioning states used for phasing updates in each run, and the *y*-axis shows the discordance between best-guess imputed genotypes and observed Illumina genotypes. Although the best-guess imputation metric is an overly simplistic way of comparing accuracy, as we emphasized in the Results section, it does tend to provide reliable rankings of different approaches and is convenient for these high-dimensional results.

In this dataset, increasing the sample size always improved imputation accuracy, as did increasing the number of conditioning states (subject to the stochastic variation of a small dataset). However, the latter approach suffered diminishing returns – for datasets of all sizes, little accuracy was gained beyond 100 conditioning states. Hence, increasing the number of conditioning states k is not an effective way to improve imputation accuracy since the running time scales quadratically with k while accuracy quickly plateaus. Changing the number of burn-in iterations had no discernable impact on accuracy, implying that the algorithm may need very few iterations to reach an effective starting point. Increasing the number of main iterations increased accuracy slightly and decreased the variance between comparable runs, but the size of this effect was quite small. The fraction of burn-in iterations based on informed conditioning updates did not show any systematic effect on accuracy.

Aside from illustrating the effects of various parameter settings on the performance of IMPUTE v2, Figure S1 also shows the effects of using the informed conditioning state approximation for phasing updates rather than no approximation at all. If running time were not an issue, we would use all K available states (chromosomes) for each phasing update, subject to the constraints outlined in the Methods section. (For example, $K = 120 + 2 \times 918 + 2 \times 458 = 2{,}872$ for individuals in the study sample and $K = 120 + 2 \times 917 = 1{,}954$ for individuals in the diploid reference panel.) Due to computational limitations, however, we typically use only the k states that are "closest" to the individual being updated. One appealing feature of this approximation is that, as k approaches K, the subset approximation converges to the full conditional model. Consequently, we would expect each accuracy curve in Figure S1 to eventually reach an asymptote (with increasing k) at the level that would be achieved with full conditional modeling. The curves in the figure seem to be close to this asymptote by k=240, suggesting that the informed subset approximation achieves nearly the accuracy of full conditional modeling while requiring much less computational effort.

On the whole, these findings show that IMPUTE v2 is not sensitive to most differences in input parameters, and that near-maximal accuracy can therefore be achieved with computationally favorable settings. Computationally rigorous settings are always desirable when resources allow, but these results provide useful guidance about which settings could be curtailed to gain speed when necessary. Finally, despite inconclusive results (which were not clarified by analysis of datasets encompassing larger regions; data not shown) about the best way to apportion burn-in iterations between conditioning methods, we still believe that it is good practice to choose random

conditioning states for at least the first few burn-in iterations and then switch to informed conditioning states for additional burn-in.

Convergence of IMPUTE v2 algorithm

One encouraging observation from Figure S1 is that, for a given *k* and sample size, IMPUTE v2 attains similar accuracy levels across more than two dozen independent runs (three burn-in iteration settings, three proportions of informed burn-in updates, and three main iteration settings, for a total of 27 runs per *k* and sample size), subject only to slight variations with the number of iterations. This consistency across runs suggests that the algorithm can integrate over parameter space quickly and thoroughly; in this case, the unknown phase of the observed genotypes is the nuisance parameter that is being integrated out. Hence, it seems that there is little risk that any particular run of the algorithm, on any reasonable setting, will get stuck in a local mode of the posterior distribution and yield drastically sub-par imputation results.

Despite these positive results, it is good practice to assess the convergence properties of the algorithm more formally. To do so, we performed additional runs on the 2 Mb region of our Scenario B dataset that was described above. Using the full sample, we ran IMPUTE v2 under all possible combinations of the following settings: 10, 25, and 100 burn-in iterations; 20, 50, and 100 main iterations; and 30 and 100 conditioning states (*k*) per phasing update. We set the first 25% of burn-in iterations to use *k* random conditioning states for phasing updates and all subsequent iterations to use *k* informed states.

For each combination of parameter settings, we conducted 10 independent runs of the algorithm; each run started with a unique, random phasing of the observed data. We separately assessed the convergence of the marginal posterior distribution of each imputed genotype using the $\hat{R}$ metric, which is a common statistic for measuring convergence in MCMC applications[1]. Informally, $\hat{R}$ compares the variance of a scalar estimand ψ between runs to the variance within each run. The intuition is that the within-run variance will be smaller than the between-run variance until approximate convergence is reached. The $\hat{R}$ metric represents the "potential scale reduction" of the distribution for ψ that might be achieved if the algorithm were run for a very large number of iterations. Values near 1 denote approximate convergence, whereas values above 1 suggest that inference could be improved with larger numbers of iterations.

IMPUTE v2 produces a probability triple, not a scalar estimand, for each imputed genotype at each iteration. To transform this output into a form that could be used with the $\hat{R}$ statistic, we converted each probability triple to an expected genotype count, or "dosage"; the expected count for a genotype with probability vector (p_0, p_1, p_2) is $\sum_{g=0}^{2} g \times p_g$, with possible values falling between 0 and 2. While the $\hat{R}$ statistic is meant to be used with normally distributed estimands, the distribution of expected genotype counts cannot formally follow a normal distribution. Nonetheless, our experience suggests that the standard interpretation of $\hat{R}$ is still reasonable in this situation.

In the 2 Mb region that we analyzed, there were over 300 common SNPs (MAF >= 5%) but only 24 rare SNPs. We separately monitored convergence at all rare SNPs and at a random subset of 100 common SNPs. At the common SNPs, we assessed the expected genotype count of each study sample individual (out of 459) as a separate scalar

estimand; at each rare SNP, we assessed only the individuals who carried copies of the minor allele (typically in heterozygous genotypes). In total, we measured convergence for 45,900 common SNP genotypes and 732 rare SNP genotypes.

The results of this analysis are shown in Table S1. The results for *k*=30 and *k*=100 were similar, so the table includes only the results for *k*=30. Here, we summarize the distribution of $\hat{R}$ values for each combination of parameter settings by the percentage of genotypes for which $\hat{R}$ exceeded 1.02 after the specified number of iterations; this is the percentage of genotypes for which the inference has not fully converged at the $\hat{R}$ = 1.02 level. The results for common and rare SNPs are shown on the left and right, respectively.

One observation from Table S1 is that convergence is largely insensitive to the number of burn-in iterations, as we concluded above. On the other hand, convergence improves when the algorithm is allowed to continue for a larger number of main iterations. The first observation suggests that the IMPUTE v2 algorithm moves around the space of observed-data haplotype reconstructions very efficiently: even starting from a random phasing of the data, it can apparently reach the posterior distribution in 10 iterations or fewer. Conversely, it may take up to 100 main iterations to fully integrate over the phase uncertainty in the data following burn-in; the implication is that independent runs with fewer main iterations might arrive at different sets of imputed genotypes.

Note, however, that our convergence threshold of $\hat{R}$ < 1.02 is very strict by most standards. If we instead used a threshold of 1.05, the average percentages in Table 4 would be 0.55%, 0.05%, and 0% for common SNPs and 0.27%, 0.05%, and 0% for rare

SNPs (with 20, 50, and 100 main iterations, respectively). These convergence levels are excellent in practical terms, and they imply that IMPUTE v2 will yield reliable and accurate results at the parameter settings used in the main study (10 burn-in iterations, followed by 20 main iterations).

Limits of informed conditioning approximation

Apart from our new way of accounting for the structure of imputation datasets, another innovation of this work is our proposed approximation to the conditional distributions used for phasing updates. One theoretical concern about this "informed" state selection method is that its accuracy might be expected to deteriorate as the amount of recombination in the dataset increases. This is because, in a given region of the genome, the concept of "k closest" genealogical neighbors loses meaning as recombination events accumulate and disrupt tree topologies. Intuitively, our approximation ought to depend on the total number of individuals in the dataset N, the number of conditioning states k, and the population-scaled recombination rate ρ for the region of interest. All other things being equal, larger N should make the approximation better by increasing the rate of coalescence; larger k should make it worse by allowing the conditioning states to have more distant common ancestors; and larger ρ should make it worse by generating more topological changes in the genealogy.

Despite the theoretical possibility that our state selection approximation could break down under certain conditions, this does not appear to be a problem in realistic applications. For example, we performed a series of imputation analyses in settings similar to the current study ($N = 1500$, $k = 120$) and saw no signs of a drop in accuracy

for regions spanning up to 20 cM on the genetic map, or ~20 Mb on the physical map (data not shown). In practice, we typically restrict imputation runs to regions of 10 Mb or less to take advantage of parallel computing capacity. These considerations, along with the apparent robustness of the method to different parameter settings, lead us to believe that our informed state selection method is a broadly applicable way of improving accuracy in imputation and phasing analyses. We also believe that this approximation could help protect against – and even benefit from – population structure in a dataset; we will pursue this idea elsewhere.

Integrating genotypes from two SNP chips

To create a diploid reference panel in Scenario B, we combined data from two SNP chips (the Affymetrix 500K chip and the Illumina 550K chip) on a common set of individuals. We began by discarding the relatively small number of SNPs that were not represented in the CEU HapMap panel since these violate the hierarchical arrangement depicted in Figure 2. Among the remaining SNPs, most were typed only on one chip, and we filtered these according to the specifications of the WTCCC[2]. We treated SNPs typed on both chips as follows: First, we combined the called genotypes. If a genotype was uncalled on both chips, we marked it 'missing'; if a genotype was called on one chip but not the other, we kept the available call; if a genotype was called on both chips and they agreed, we kept the call; if a genotype was called on both chips and they disagreed, we marked the genotype 'missing'. We then applied the same SNP filters as before to these consensus genotypes, except that we used a more stringent missing data filter of 1% (in contrast to the 5% filter used by the WTCCC). This eliminated SNPs that had many

disparate calls between chips or were difficult to call on both chips. Few of the SNPs that survived this filtering regime would have been removed by applying the standard filters to either chip separately, and almost none would have been removed from both chips in this way (data not shown) – this gives us confidence that the resulting combined dataset is of high quality.

Association testing of cases imputed from controls

One manifestation of Scenario B, which we outlined in the Introduction, entails using a large set of controls genotyped on multiple SNP chips to impute case cohorts genotyped on only one of those chips. This study design is currently being applied by the WTCCC, and it may soon be used in other studies as well.

One potential concern about this design is that false positive associations could arise at SNPs where the control genotypes are observed but the case genotypes are imputed. Specifically, SNPs that are hard to impute will have lower "quality" in cases (i.e., the imputed genotypes will be known with less certainty), and it is well known that false positives can arise in the analogous situation of differential data quality when all genotypes have been assayed directly[3]. One proposed solution in that context is to use "fuzzy" genotype calls in association tests rather than marking uncertain genotypes as missing[4]. We believe that taking a similar approach in our example – i.e., using association tests that properly account for any uncertainty in the genotype calls, whether imputed or assayed – should prevent artificially inflated levels of false positives.

To test this proposition empirically, we used the control genotypes from the current study to impute genotypes in another control cohort (the United Kingdom Blood

Service controls, or UKBS) from the WTCCC study[2]. The filtered 1958 Birth Cohort (58C) control dataset includes 1,377 individuals genotyped on both the Affymetrix 500K and Illumina 550K SNP chips, while the filtered UKBS dataset includes 1,458 individuals genotyped on only the Affymetrix 500K chip. The 58C dataset for chromosome 10 comprises an integrated panel of 44,875 SNPs (as detailed above), of which 22,219 are not represented on the Affymetrix chip. We used IMPUTE v2 to impute the Illumina-only SNPs in the UKBS data from a combined reference panel of HapMap CEU haplotypes and 58C genotypes. We ran IMPUTE v2 with the same settings used in the main study: k=40 and k=80; 10 burn-in iterations; 20 main iterations.

We evaluated the false positive rate by performing frequentist association tests between observed 58C genotypes and imputed UKBS genotypes under an additive model. To account for the uncertainty in the imputed genotypes, we used a likelihood-based procedure that integrates over the distribution of the missing data[5] and is implemented in our SNPTEST software. We plotted the resulting p-p plots for IMPUTE v2 with k=40 in Figure S2; the plots for k=80 were almost identical, so we have not shown them.

Figure S2A shows the p-values for all common (MAF >= 5%) SNPs, and Figure S2B shows the p-values for all rare SNPs; allele frequencies were calculated in the 58C dataset since the true UKBS genotypes are unknown. Each plot includes a 95% "concentration band"[2], which does not have a simple statistical interpretation but does show the approximate spread that null p-values might exhibit. Both plots show slight deviations from the $y = x$ line (red), but the p-values stay mostly within the concentration band and are well-behaved in the tail of the distribution. We also note that the limited

deviation seen here is consistent with the small differences between the 58C and UKBS at SNPs typed in both control sets.

These results show that imputing cases from controls, then testing for differences between the imputed case genotypes and the observed control genotypes, will not necessarily inflate the false positive rate if appropriate association tests are carried out. We strongly caution, however, that association tests that do not account for the uncertainty of imputed genotypes could perform badly in this setting.

REFERENCES

1. Gelman A, Carlin, J.B., Stern, H.S., Rubin, D.B. (2003) Bayesian Data Analysis. Chapman and Hall/CRC
2. The Wellcome Trust Case Control Consortium (2007) Genome-wide association study of 14,000 cases of seven common diseases and 3,000 shared controls. Nature 447:661-678
3. Clayton DG, Walker NM, Smyth DJ, Pask R, Cooper JD, Maier LM, Smink LJ, Lam AC, Ovington NR, Stevens HE, Nutland S, Howson JM, Faham M, Moorhead M, Jones HB, Falkowski M, Hardenbol P, Willis TD, Todd JA (2005) Population structure, differential bias and genomic control in a large-scale, case-control association study. Nat Genet 37:1243-1246
4. Plagnol V, Cooper JD, Todd JA, Clayton DG (2007) A method to address differential bias in genotyping in large-scale association studies. PLoS Genet 3:e74
